# Interactive effects of aging and aerobic capacity on energy metabolism–related metabolites of serum, skeletal muscle, and white adipose tissue

**DOI:** 10.1007/s11357-021-00387-1

**Published:** 2021-06-05

**Authors:** Haihui Zhuang, Sira Karvinen, Timo Törmäkangas, Xiaobo Zhang, Xiaowei Ojanen, Vidya Velagapudi, Markku Alen, Steven L. Britton, Lauren G. Koch, Heikki Kainulainen, Sulin Cheng, Petri Wiklund

**Affiliations:** 1 School of Life Sciences and Biotechnology, Shanghai Jiao Tong University, Shanghai, China; 2 Key Laboratory of Systems Biomedicine (Ministry of Education), and Exercise Translational Medicine Center, Shanghai Center for Systems Biomedicine, Shanghai Jiao Tong University, Shanghai, China; 3 Faculty of Sport and Health Sciences, University of Jyväskylä, Jyväskylä, Finland; 4 Metabolomics Unit, Institute for Molecular Medicine Finland (FIMM), HiLIFE, University of Helsinki, Helsinki, Finland; 5 Department of Medical Rehabilitation, Oulu University Hospital, Oulu, Finland; 6 Department of Anesthesiology, University of Michigan, Ann Arbor, MI USA; 7 Molecular and Integrative Physiology, University of Michigan, Ann Arbor, MI USA; 8 Department of Physiology and Pharmacology, The University of Toledo College of Medicine and Life Sciences, Toledo, OH USA; 9 Huawei Helsinki Research Center, Huawei Technologies Oy (Finland) Co. Ltd, Helsinki, Finland

**Keywords:** Aerobic capacity, Aging, Metabolomics, Metabolites

## Abstract

Aerobic capacity is a strong predictor of longevity. With aging, aerobic capacity decreases concomitantly with changes in whole body metabolism leading to increased disease risk. To address the role of aerobic capacity, aging, and their interaction on metabolism, we utilized rat models selectively bred for low and high intrinsic aerobic capacity (LCRs/HCRs) and compared the metabolomics of serum, muscle, and white adipose tissue (WAT) at two time points: Young rats were sacrificed at 9 months of age, and old rats were sacrificed at 21 months of age. Targeted and semi-quantitative metabolomics analysis was performed on the ultra-pressure liquid chromatography tandem mass spectrometry (UPLC-MS) platform. The effects of aerobic capacity, aging, and their interaction were studied via regression analysis. Our results showed that high aerobic capacity is associated with an accumulation of isovalerylcarnitine in muscle and serum at rest, which is likely due to more efficient leucine catabolism in muscle. With aging, several amino acids were downregulated in muscle, indicating more efficient amino acid metabolism, whereas in WAT less efficient amino acid metabolism and decreased mitochondrial β-oxidation were observed. Our results further revealed that high aerobic capacity and aging interactively affect lipid metabolism in muscle and WAT, possibly combating unfavorable aging-related changes in whole body metabolism. Our results highlight the significant role of WAT metabolism for healthy aging.

**Supplementary Information:**

The online version contains supplementary material available at 10.1007/s11357-021-00387-1.

## Introduction

Epidemiological studies have indicated that low aerobic capacity, expressed as maximal oxygen uptake (VO_2max_), is a major risk factor for cardio-metabolic diseases [1], whereas high aerobic capacity is associated with a more favorable cardio-metabolic health profile [2, 3], even in the presence of excess adiposity [4]. These observations are complemented by multiple randomized controlled trials, which have shown that exercise training significantly improves cardiorespiratory fitness and adiposity-related cardio-metabolic biomarkers [5]. Furthermore, aerobic capacity has been shown to be a strong independent predictor of cardiovascular and all-cause mortality in many population cohorts [6]. These studies suggest there is a fundamental connection between impaired aerobic metabolism, complex disease risk, and reduced longevity.

Epidemiological studies have also shown that aerobic capacity and physical activity level decrease with age [7, 8] concomitantly with quantitative and structural changes in body composition [9]. These changes are associated with alterations in whole body metabolism, insulin resistance, low-grade inflammation, and dyslipidemia, which contribute to the development of cardio-metabolic disease with age [10, 11]. However, it is not well understood to what extent the aging-related changes in metabolic profiles are attributable to either intrinsic aerobic capacity or decreased physical activity.

To enable the investigation of intrinsic aerobic endurance capacity on metabolic profile, Koch and Britton [12] developed heterogeneous rat lines by artificial selection for low and high inborn exercise capacity. This rat model of low capacity runners (LCRs) and high capacity runners (HCRs) prospectively tests the association between aerobic exercise capacity and survivability [13]. The phenotype of HCRs is coincident with a host of health benefits [14], including a 28–40% increased lifespan [15]. A recent study in HCRs and LCRs showed that an enhancement of aerobic capacity could mitigate some of the changes in the plasma metabolic profile, which were associated with aging [16]. Concomitantly with higher aerobic capacity, muscle mitochondrial function and oxidative energy metabolism are enhanced in HCRs compared with those in LCRs [17, 18]. In addition to skeletal muscle, white adipose tissue (WAT) shares an important role in the regulation of whole body metabolism [19] and WAT-to-muscle communication is critical in overall metabolic health [20].

Most previous studies have examined the effects of aerobic capacity on metabolism only in one tissue [21, 22] and using only young animals (3-4 months of age) [17]. Thus, possible interactive effects of aging and aerobic capacity on tissue metabolism are yet to be resolved. Here, we assessed metabolic profiles in serum, muscle, and WAT in both young (9 months of age) and old (21 months of age) HCR and LCR groups by using targeted and semi-quantitative metabolomics analysis which was performed on the triple quadrupole tandem mass spectrometry coupled to the ultra-pressure liquid chromatography (UPLC-MS) platform. We propose to define the most prominent differences for aerobic capacity in the metabolic profile of skeletal muscle [17, 18, 23, 24]. Also, we expect that aerobic capacity and aging interactively affect several metabolites in muscle and WAT leading to changes in serum metabolite levels, as LCRs are known to have higher metabolic disease risk and shorter lifespan [13, 14].

## Materials and methods

### Animal model

The HCR/LCR rat model was derived from a genetically heterogeneous founder population (N:NIH stock) and bred with two-way artificial selection [12]. Animals were evaluated at 11 weeks of age for maximal running capacity at the University of Michigan (Ann Arbor, MI, USA) with a speed-ramped treadmill running test (15° slope, initial velocity of 10 m/min, increased 1 m/min every 2 min). In this study, 34 female rats (16 HCRs and 18 LCRs) from generations 23–27 of selection were used. All rats were kept in an environmentally controlled facility with light/dark cycle of 12/12 h and had free access to food and tap water (R36, Labfor, Stockholm, Sweden).

### Testing procedure

After arriving to Finland, rats were tested for maximal running capacity at the age of 9 months with the same speed-ramped running test as described previously [18]. Subsequently, both HCRs and LCRs were divided into weight and maximal running capacity–matched sub-groups: HCR-Y or LCR-Y (young, *n* = 10 in each group), and HCR-O (old, *n* = 6) or LCR-O (old, *n* = 8). After maximal aerobic capacity testing, HCR-Y and LCR-Y were weighed and sacrificed and hindlimb skeletal muscles, WAT, and blood samples were collected. HCR-O and LCR-O were continued to be housed individually in a standard cage until the age of 21 months. Maximal aerobic capacity was assessed again at the age of 21 months. After testing, animals were weighed and sacrificed and skeletal muscle, WAT, and serum samples were harvested.

### Tissue collection

Soleus, extensor digitorum longus (EDL), plantaris, gastrocnemius, quadriceps femoris muscle, adipose tissue around the ovaries, visceral adipose tissue, and retroperitoneal adipose tissue were excised and weighed. Tissue samples were snap frozen in liquid nitrogen and then stored in −80°C until analyses. Gastrocnemius, retroperitoneal adipose tissue, and serum were used for metabolomics analyses. Skeletal muscle mass was calculated as the sum weight of soleus, EDL, plantaris, gastrocnemius, and quadriceps femoris.

### Metabolomics analyses

Targeted and semi-quantitative metabolomics analyses were performed on a Waters Xevo TQ-S triple quadrupole tandem mass spectrometer coupled to the ultra-pressure liquid chromatography (UPLC-MS) platform using the previously published protocol in FIMM (Institute for Molecular Medicine Finland) [25]. Briefly, metabolites were extracted from 100 μL serum, 20 mg muscle, and 20 mg WAT samples respectively using protein precipitation by adding acetonitrile +1% formic acid. The collected extracts were dispensed in Ostro 96-well plates (Waters Corporation, Milford, USA) and filtered by applying a vacuum at a delta pressure of 300–400 mbar for 2.5 min on robot’s vacuum station. Filtered sample extract (5 μL) was injected in an Acquity UPLC system coupled to a Xevo TQ-S triple quadrupole mass spectrometer (Waters Corporation, Milford, MA, USA) which was operated in both positive and negative polarities with switching time of 20 ms. Multiple Reaction Monitoring (MRM) acquisition mode was selected for the quantification of metabolites. MassLynx 4.1 software was used for data acquisition, data handling, and instrument control. Data processing was done using TargetLynx 4.1 software. Eighty-nine metabolites in serum, 71 in muscle, and 71 in WAT were identified in whole samples.

### Statistical analysis

The descriptive characteristics of rats were analyzed by using IBM SPSS Statistics 24.0 (SPSS, Chicago, USA). Data were checked for normality with the Shapiro-Wilk test. As most of the variables were not normally distributed, nonparametric tests were chosen for group comparisons, and statistical significance was set at *p* < 0.05. The metabolomics analysis was same as in our previous study with serum and muscle samples [26].

All metabolomics data were transformed with a suitable transformation from the Box-Cox family and then scaled by dividing each variable with its standard deviation (unit-variance scaling, criteria for absolute value of the residuals for skew and kurtosis < 2). We used partial-least-squares discriminant analysis (PLS-DA) for age and aerobic capacity group comparisons for assessing multivariate metabolite profiles. Permutation tests (G = 200 per model) were used to validate the model and to avoid over-fitting.

We also performed comparative univariate analyses using linear regression models to assess (1) age (old vs. young) and aerobic capacity (high vs. low) group differences on metabolites, and (2) associations between metabolites and running speed while accounting also for the grouping structure (age and aerobic capacity). In the former case, we computed the *p* value of the joint impact of both grouping factors (age and aerobic capacity) and their interaction. In the latter case, we used the *p* value for the joint contribution of the metabolite-related predictors in the model (metabolite, metabolite × age, metabolite × aerobic capacity, metabolite × age × aerobic capacity). Model equations are shown in the Technical Supplement. In a PLS model, variable importance in projection (VIP) is used as an index of the estimated importance of a variable as a contributor to group separation in the model. Among metabolites, we selected metabolites based on VIP values ≥ 1 and *p* < 0.05 for the model combined impact of the factors in the univariate linear model.

For running speed-metabolite association, we considered as influential metabolites those with FDR-corrected *p* value of the association with metabolite < 0.05. Multivariate and univariate modeling were performed using a custom script in the R programming environment, version 4.0.2, utilizing packages psych (version 2.0.8) for assessment of normality of residuals and emmeans (version 1.5.1, https://CRAN.R-project.org/package=emmeans.) for computing estimated marginal coefficients and rolls (version 1.20.0) for PLS models [27].

### Pathway analysis

MetaboAnalyst (v3.5) was used for pathway enrichment analysis to map significantly differential metabolites found in PLS-DA and regression models to their corresponding pathways [28, 29]. This web-based tool relies on the knowledgebase of Kyoto Encyclopedia of Genes and Genomes (KEGG) metabolic pathway.

## Results

### Body mass, running capacity, and skeletal muscle mass/body mass ratio

**Fig. 1:**
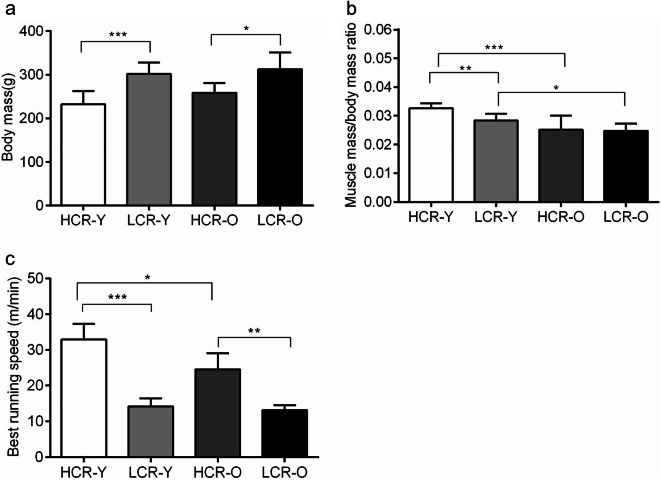
The effects of aerobic capacity and aging on muscle mass to body mass ratio and maximal running speed in rats. Body mass (**a**), muscle mass to body mass ratio (**b**), and maximal running speed (**c**) in the studied rat groups. HCR-Y, high capacity runner, young; LCR-Y, low capacity runner, young; HCR-O, high capacity runner, old; LCR-O, low capacity runner, old. Data is presented as mean ± SD. *p <0.05, **p <0.01, and ***p<0.001

HCRs had lower body mass and higher maximal running speed than LCRs both at 9 months (*p* < 0.001) and 21 months of age (*p* = 0.002, *p* = 0.006, respectively) (Fig. 1a, c). Hindlimb skeletal muscle mass to body mass ratio was lower in both HCRs and LCRs when comparing 21-month-old rats to 9-month-old (Fig. 1b). The maximal running speed was 25.5% lower in 21-month-old HCRs compared with 9-month-old HCRs (*p* < 0.001) whereas in LCRs, no significant difference in running speed with age was observed (*p* = 0.360; Fig. 1c).

### Effects of intrinsic aerobic capacity and aging on metabolic profiles of serum, muscle, and white adipose tissue

**Fig. 2:**
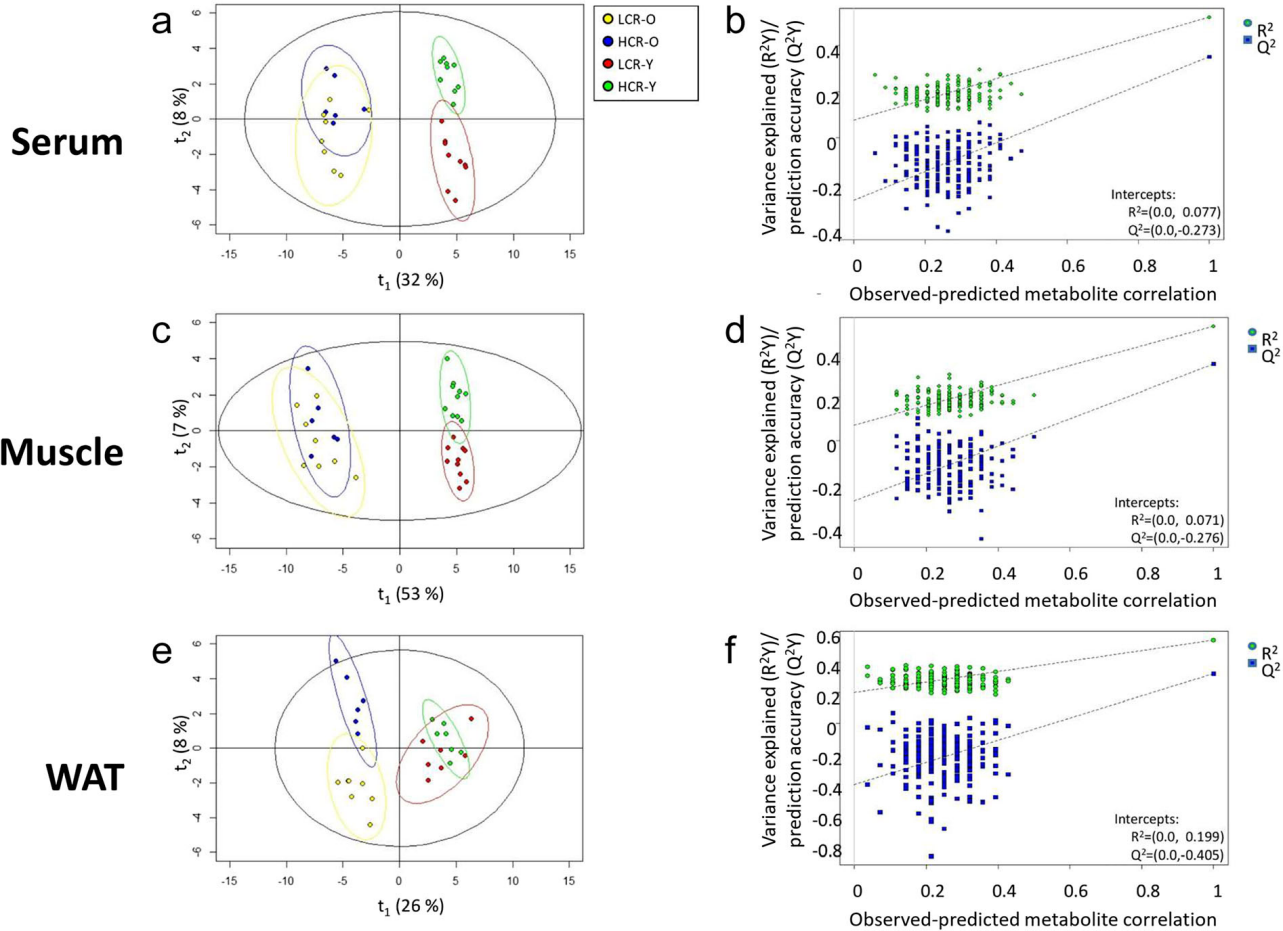
Score plot of PLS-DA in serum (**a**), muscle (**c**), and WAT (**e**) with aerobic capacity and age and linear regression model with 200 times permutation test of the models for serum (**b**), muscle (**d**), and WAT (**f**). LCR-O, low capacity runner, old; HCR-O, high capacity runner, old; LCR-Y, low capacity runner, young; HCR-Y, high capacity runner, young

We used PLS-DA to identify differences in the metabolic profiles between the rat lines (LCR vs. HCR) and age groups (young, Y vs. old, O). There were clear separations in serum (*Q*^2^ = 0.443, *Q*^2^ intercept = −0.273), muscle (*Q*^2^ = 0.350, *Q*^2^ intercept = −0.276), and WAT (*Q*^2^ = 0.325, *Q*^2^ intercept = −0.405) metabolic profiles (Fig. 2; Table S1). However, a large difference between *R*^2^ and *Q*^2^ was observed in WAT tissue, suggesting a poor predictive performance of the model in WAT (Table S1). The corresponding associations between running speed and metabolites in groups of aerobic capacity and age are shown in Supplementary Table S4. When expressing the data via running speed, the metabolic profiles of serum and muscle are similar as with aerobic capacity, but the profile of WAT separated also at young age (Fig. S1).

### Effects of intrinsic aerobic capacity and aging on single metabolites of serum, muscle, and white adipose tissue

**Fig. 3:**
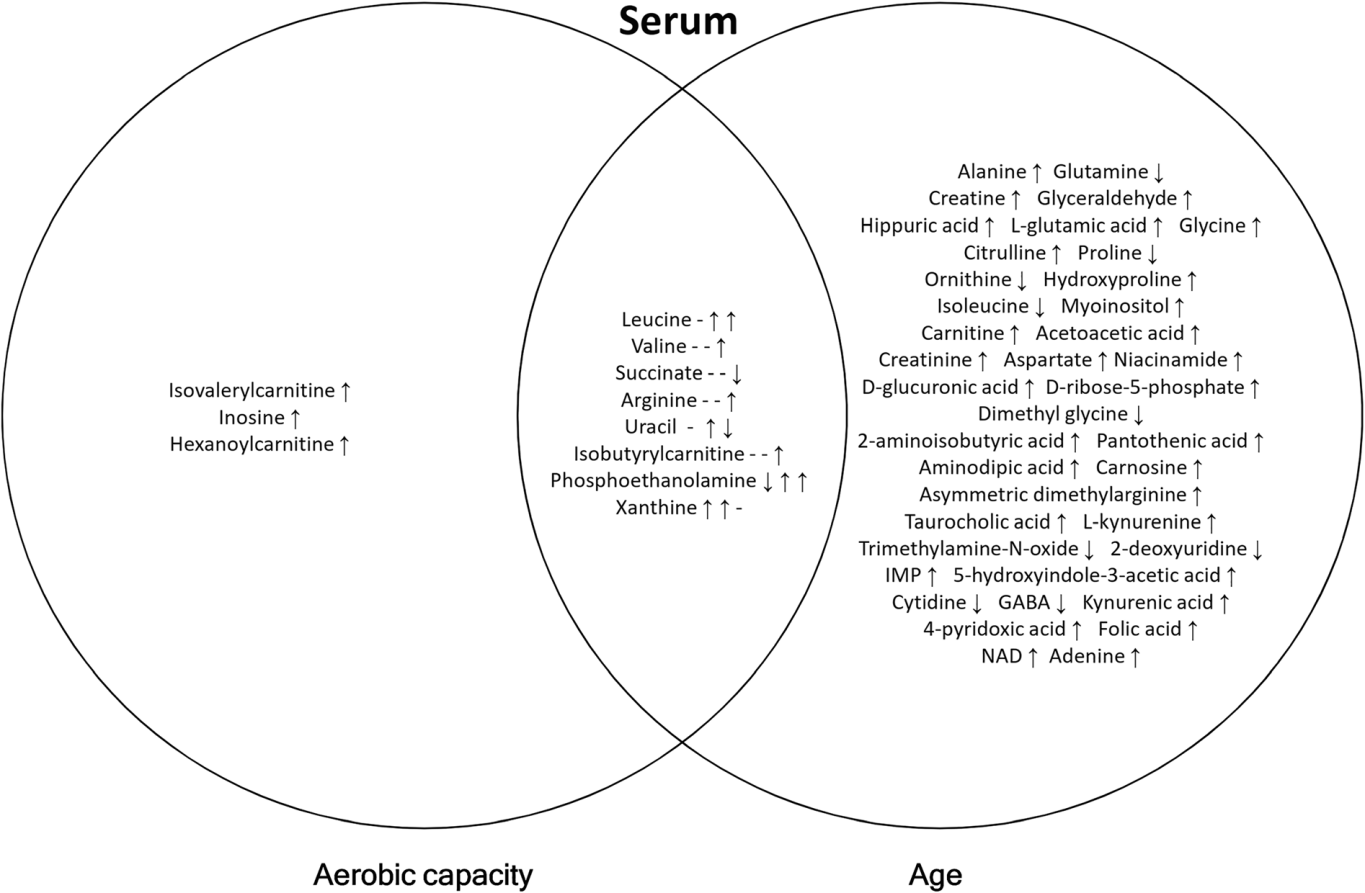
Venn diagram of differentially expressed metabolites with aerobic capacity, age, and their interaction in serum. Arrows represent the direction of regression coefficient; that in the overlapping region (aerobic capacity * age) may originate from aerobic capacity (1st arrow) and age (2nd arrow) or their interaction (3rd arrow)

**Fig. 4:**
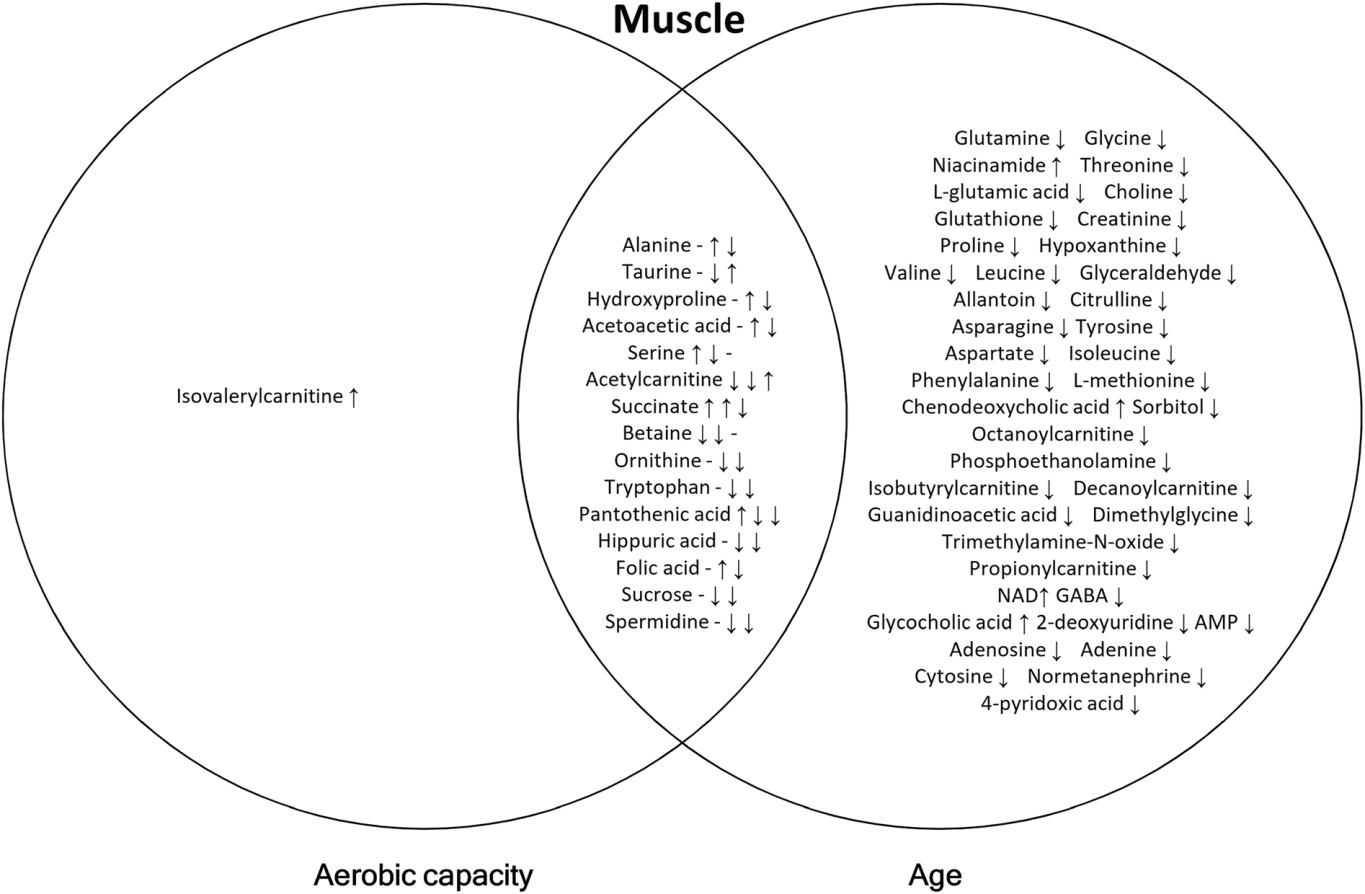
Venn diagram of differentially expressed metabolites with aerobic capacity, age, and their interaction in muscle. Arrows represent the direction of regression coefficient; that in the overlapping region (aerobic capacity * age) may originate from aerobic capacity (1st arrow) and age (2nd arrow) or their interaction (3rd arrow) or both

**Fig. 5:**
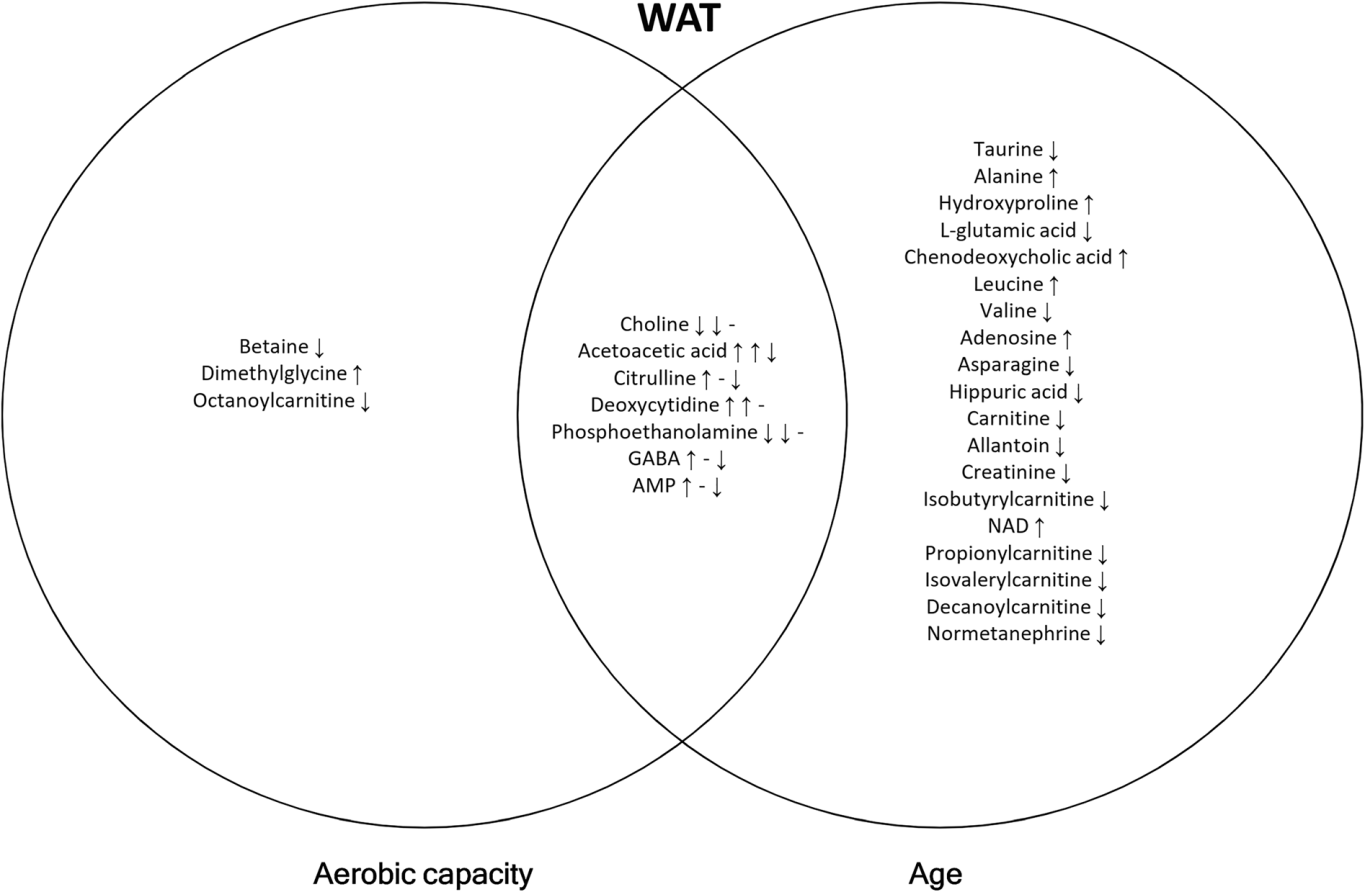
Venn diagram of differentially expressed metabolites with aerobic capacity, age, and their interaction in WAT. Arrows represent the direction of regression coefficient; that in the overlapping region (aerobic capacity * age) may originate from aerobic capacity (1st arrow) and age (2nd arrow) or their interaction (3rd arrow)

Next, we compared the differences at the level of single metabolites between HCRs and LCRs with univariate analysis. The Venn diagrams of single metabolites changed by aerobic capacity, aging, and their interaction in serum, muscle, and WAT are shown in Figs. 3, 4, and 5. The corresponding univariate analyses with regression coefficients are shown in Supplementary Table S2 and design-controlled skew and kurtosis estimates for metabolites after most optimal transformation in Supplementary Table S5. Supplementary Table S3 shows the pathway analysis of the significantly changed metabolites.

#### Effect of intrinsic aerobic capacity on serum, muscle, and WAT metabolites

In serum, isovalerylcarnitine, inosine, and hexanoylcarnitine were upregulated with high aerobic capacity, whereas in muscle, only isovalerylcarnitine was upregulated (Figs 3 and 4; Table S2). In WAT, high aerobic capacity upregulated the level of dimethylglycine and downregulated the levels of betaine and octanoylcarnitine (Fig. 5; Table S2). The predominant pathways that were affected by aerobic capacity were nucleotide metabolism in serum and amino acid metabolism in WAT (*p*<0.050; Table S3).

#### Effect of aging on serum, muscle, and WAT metabolites

In serum, aging was associated with higher levels of several metabolites, having the most prominent effect on amino acid metabolites (Fig. 3; Table S2). The levels of isoleucine, proline, ornithine, and glutamine were downregulated, while alanine glycine citrulline, hydroxyproline, and aspartate were upregulated in serum (Fig. 3). In muscle, several amino acids were downregulated including branched-chain amino acids (BCAAs; valine, leucine, and isoleucine) (Fig. 4; Table S2). In WAT, alanine and leucine levels were upregulated and carnitine and several acylcarnitines (isobutyrylcarnitine, propionylcarnitine, and isovalerylcarnitine) were downregulated (Fig. 5; Table S2). The predominant pathways that were affected by aging were linked to amino acid metabolism in serum, muscle, and WAT (*p*≤0.050; Table S3).

#### Effect of the interaction of aerobic capacity and aging on serum, muscle, and WAT metabolites

We found that high aerobic capacity and aging interactively upregulated the levels of leucine, valine, arginine, isobutyrylcarnitine, and phosphoethanolamine, and downregulated succinate and uracil in serum (Fig. 3; Table S2). In muscle, acetylcarnitine and taurine were upregulated, whereas alanine, hydroxyproline, acetoacetic acid, succinate, ornithine, tryptophan, pantothenic acid, hippuric acid, folic acid, sucrose, and spermidine were downregulated (Fig. 4; Table S2). In WAT, aerobic capacity and aging interactively downregulated acetoacetic acid, citrulline, GABA, and AMP (Fig. 5; Table S2). The predominant pathways that were affected by the interaction of aerobic capacity and aging were linked to amino acid metabolism in serum and muscle and lipid metabolism in WAT (*p*<0.050; Table S3).

## Discussion

The present study suggests that aerobic capacity and aging have different impacts on the metabolic profiles of serum, skeletal muscle, and white adipose tissue (WAT). Contrary to our hypothesis, only isovalerylcarnitine was upregulated by high aerobic capacity in muscle, whereas aging changed a large amount of metabolites predominantly linked to amino acid metabolism in serum, muscle, and WAT. Aerobic capacity and aging interactively affected several metabolites, the most predominant pathways being amino acid metabolism in serum and muscle and lipid metabolism in WAT.

### Aerobic capacity and aging have distinct effects on metabolic profiles of muscle and WAT

We observed a clear separation of the metabolic profiles (PLS-DA) of serum and muscle in HCRs and LCRs at young age, whereas at old age, the metabolic profiles overlapped (HCR-Y vs. LCR-Y and HCR-O vs. LCR-O; Fig. 2). Interestingly, the opposite was observed for WAT, where the metabolic profiles overlapped at young age and showed a clear separation between HCRs and LCRs at old age (Fig. 2e). A previous study by Falegan et al. examined the metabolic profile of plasma of young (13 months) and old (26 months) male HCR and LCR rats and observed a strong profile separation in old and LCRs, whereas young and HCRs were found less predictive [16]. Hence, they concluded that in plasma, metabolomics analysis better predicts age rather than aerobic capacity. Yet our results from serum suggest that at old age, the metabolic profiles of HCRs and LCRs are more similar. The reason for the divergence in observations may be due to differences in sex, age, and generation (Falegan et al. used rats from generations 17 and 19 of selection), fasting duration, and euthanasia method. Different metabolomics platforms may also account for the discrepant results as Falegan et al. used NMR proton spectrometry, while in the present study we used UPLC-MS in targeted metabolite profiling. Nevertheless, our results highlight the role of WAT metabolism in healthy aging.

We further observed that the metabolic profiles of serum and muscle were similar irrespective of whether the data was expressed using the rat lines and age groups (Fig. 2a, c) or via running speed (Fig. S1a, c). Our result suggests that the separation of the rat lines through maximal running capacity has driven the changes in the metabolism of these rats, as high running speed is the practical manifestation of the genetic breeding of HCR rats and LCR rats. Interestingly, unlike in the line vs. age comparison, the metabolic profiles of young rats appeared differentially expressed when shown via running speed (Fig. S1e). This observation may be due to a large difference in the running speed between the rat lines at young age (HCR-Y vs. LCR-Y), but that is less prominent at old age (Fig. 1c). These observations suggest that aerobic capacity enhances the metabolism of adipose tissue and supports earlier studies which have shown that exercise training enhances white adipose tissue metabolism in HCR and LCR rats [30], and that adipose tissue may mediate some of the health benefits of aerobic exercise training [31, 32].

### High aerobic capacity is associated with efficient leucine catabolism in muscle and altered amino acid and fatty acid metabolism in WAT

Skeletal muscle is an important tissue contributing both to aerobic capacity and whole body metabolism [33]. Hence, it was unexpected that high aerobic capacity was associated only with upregulation of isovalerylcarnitine in muscle (Fig. 3). Isovalerylcarnitine is produced during leucine catabolism and accumulation of isovalerylcarnitine in serum might indicate a defect in leucine catabolism [34, 35]. In humans, increased concentrations of isovalerylcarnitine and hexanoylcarnitine have been previously reported in type 2 diabetes [36–38] as well as in a model of type 2 diabetes-Zucker diabetic fatty rats [39]. However, efficient leucine catabolism may also lead to accumulation of isovalerylcarnitine when energy demand decreases. HCRs may catabolize leucine more actively during movement to supply acetyl-coenzyme A to tricarboxylic acid cycle, whereas at rest the produced isovaleryl-coenzyme A is not needed, and is further metabolized to isovalerylcarnitine. Supporting this hypothesis, our previous studies have shown increased expression of the enzymes of BCAA catabolism in skeletal muscle of HCRs [24] and physically active humans [40]. Also, a previous study by Overmyer et al. showed that young (3–4.5 months of age) HCRs oxidize BCAAs (leucine, isoleucine, and valine) more efficiently compared to LCRs of the same age [17]. Furthermore, they found that muscle BCAAs were lower in HCRs than in LCRs after 10 min of speed-ramped treadmill running. These changes were paralleled by a fall in plasma BCAAs in HCRs, indicating increased utilization of BCAAs during exercise. According to our results, we speculate that during resting at fasted state, HCRs may accumulate isovalerylcarnitine into muscle and serum due to more efficient leucine catabolism in muscle.

Comparedwith other metabolically active tissues, the oxidative capacity of WAT is relatively low. For instance, O_2_ consumption per kg wet weight in WAT is about one-tenth of that of the resting skeletal muscle [41]. In WAT tissue, high aerobic capacity upregulated dimethylglycine and downregulated betaine (trimethylglycine) (Fig. 5) suggesting increased methylation of homocysteine to methionine, which has various functions in metabolism [42]. However, the possible role of betaine in HCR WAT remains to be shown. Octanoylcarnitine was also downregulated (Fig. 5) suggesting more efficient fatty acid oxidation with higher aerobic capacity in WAT. We observed a difference only in three metabolites, indicating relatively small differences between HCR and LCR rats in WAT metabolism.

### Aging was associated with the most prominent changes in serum, muscle, and WAT metabolites

In our study setup, aging was associated with the most prominent changes in serum, muscle, and WAT (Figs. 3, 4, and 5) supporting previous findings that highlighted the importance of age when investigating metabolic changes in aerobic capacity [16]. In serum, aging was associated with higher level of several metabolites, having the most noticeable effect on amino acid metabolites (Fig. 3; Table S3). The levels of isoleucine, proline, ornithine, and glutamine were downregulated, while citrulline, alanine, glycine, hydroxyproline, and aspartate were upregulated in serum (Fig. 3). With aging, the serum concentrations of amino acids typically decrease. This is mainly related to the reduction of dietary protein intake and alterations in gluconeogenesis and the function of urea cycle [43, 44]. Upregulated citrulline and alanine levels in serum may indicate less efficient urea cycle.

In muscle, the levels of several amino acids were downregulated, including BCAAs, indicating more efficient amino acid metabolism with aging. In human, basal amino acid metabolism may be unaffected by age, yet old animals and humans appear to have a decreased ability to respond to anabolic stimuli [45, 46]. Our results suggest that certain metabolic pathways activate with aging, possibly combating the unfavorable changes in skeletal muscle metabolism and loss of muscle mass that occurs with aging [47].

In contrast, in WAT, alanine and leucine levels were upregulated indicating less efficient amino acid metabolism with aging (Fig. 5). This observation is in agreement with previous studies suggesting that adipose tissue plays a central role in the development of insulin resistance, metabolic dysfunction, inflammation, and impaired regenerative capacity during aging [48–50]. In addition, WAT carnitine and several acylcarnitines (isobutyrylcarnitine, propionylcarnitine, and isovalerylcarnitine) were downregulated with aging. Acylcarnitines transport fatty acids into mitochondria and are essential for β-oxidation and, hence, energy metabolism [51]. Downregulation of carnitine and acylcarnitines in WAT suggests a decreased mitochondrial β-oxidation, which may contribute to aging-related fat accumulation.

### Interaction of aerobic capacity and aging alters lipid metabolism in muscle and WAT

Previous human studies propose that high aerobic capacity reduces symptoms of age-related disorders, including obesity, diabetes, inflammation, and cardiovascular diseases [52, 53], suggesting that the interaction of aerobic capacity and aging has a combined effect on metabolism. In the present study, high aerobic capacity and aging interactively upregulated the levels of leucine and valine in serum (Fig. 3; Table S3), indicating less efficient BCAA utilization from serum at rest with high aerobic capacity and aging. It should be noted that during aging, aerobic capacity of HCR rats declines more than that of LCRs (Fig. 1), which may at least partially explain this interaction. In turn, in muscle, several amino acids were downregulated (alanine, hydroxyproline, ornithine, and tryptophan) (Fig. 4; Table S3), suggesting an alteration in amino acid metabolism with higher aerobic capacity and aging. Also, acetoacetic acid, succinate, and pantothenic acid linked to lipid metabolism—more precisely to acetyl-CoA formation from liver-originating acetoacetate—were downregulated in muscle, indicating that interaction of aerobic capacity and aging reduces mitochondrial energy metabolism in this tissue. Although the role of ketone bodies in skeletal muscle metabolism is limited, it has been found both in animal and human studies that ketogenic diet increases mitochondrial energy metabolism, especially fat oxidation [54, 55]. In WAT, aerobic capacity and aging interactively downregulated acetoacetic acid, citrulline, GABA, and AMP, further referring to altered lipid metabolism (Fig. 5; Table S3). Our results suggest that high aerobic capacity and advanced age interactively affect lipid metabolism in muscle and WAT, possibly combating aging-related unfavorable changes in the whole body metabolism as well as fat accumulation.

## Conclusion

Our results suggest that high aerobic capacity is associated with an accumulation of isovalerylcarnitine into muscle and serum at rest, which is likely due to more efficient leucine catabolism in muscle. Aerobic capacity also altered amino acid and fatty acid metabolism in WAT, although significant difference was observed only in three metabolites. In our study setup, aging was associated with the most prominent changes in metabolites. In muscle, several amino acids were downregulated, suggesting more efficient amino acid metabolism with aging. In contrast, the results from WAT indicated less efficient amino acid metabolism and decreased mitochondrial β-oxidation with aging. Our results further revealed that high aerobic capacity and age interact affecting lipid metabolism in muscle and WAT, possibly combating aging-related unfavorable changes in whole body metabolism as well as fat accumulation. Our results highlight the role of WAT metabolism in healthy aging. Future studies are needed to determine whether improved aerobic capacity by training could restore the observed aging-related metabolic changes.

## Supplementary information

Supplementary Figure 1 Score plot of PLS-DA in serum (a), muscle (c) and WAT (e) with running speed (y-axis) and metabolites (X-axis) accounted for age and aerobic capacity. VIP and FDP-corrected p-values are shown for serum (b), muscle (d) and WAT (f). LCR-O=low capacity runner, old; HCR-O=high capacity runner, old; LCR-Y=low capacity runner, young; HCR-Y=high capacity runner, young (PNG 1078 kb)

High Resolution (TIF 1887 kb)

Table S1 (DOCX 14 kb)

Table S2 (DOCX 40 kb)

Table S3 (DOCX 23 kb)

Table S4 (DOCX 60 kb)

Table S5 (DOCX 25 kb)

ESM 1 (DOCX 15 kb)

## Data Availability

The data set is now deposited to MetaboLights https://www.ebi.ac.uk/metabolights/MTBLS2234/descriptors.
