# Supplementary figures and images for: Interactive effects of aging and aerobic capacity on energy metabolism–related metabolites of serum, skeletal muscle, and white adipose tissue
Source: GeroScience. 2021 Jun 5;43(6):2679–91. doi: 10.1007/s11357-021-00387-1 (PMC8602622; doi:10.1007/s11357-021-00387-1)

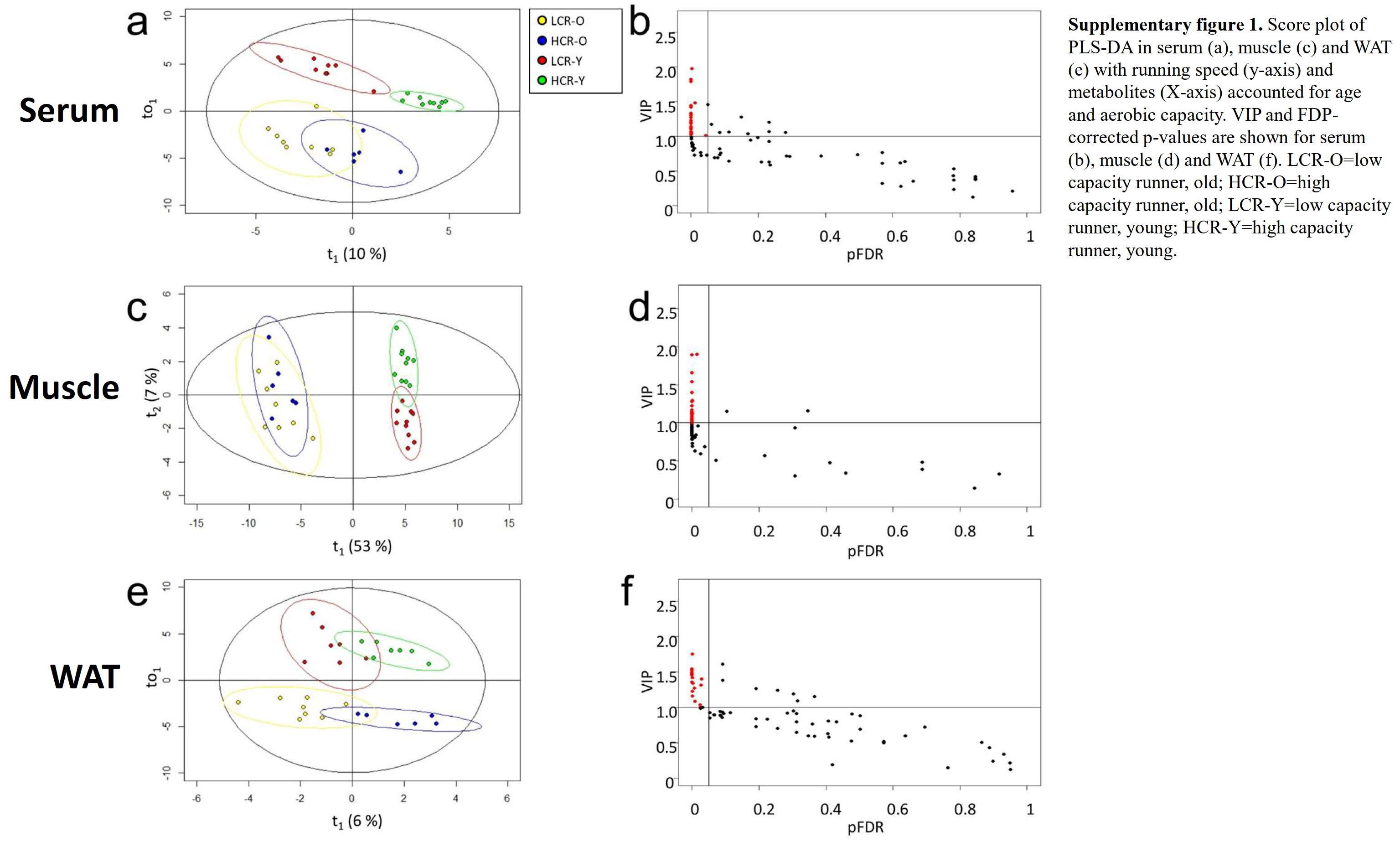

Supplement: Supplementary file 1 — Score plot of PLS-DA in serum (a), muscle (c) and WAT (e) with running speed (y-axis) and metabolites (X-axis) accounted for age and aerobic capacity. VIP and FDP-corrected p-values are shown for serum (b), muscle (d) and WAT (f). LCR-O=low capacity runner, old; HCR-O=high capacity runner, old; LCR-Y=low capacity runner, young; HCR-Y=high capacity runner, young (PNG 1078 kb) [file 11357_2021_387_Fig1_ESM.png]

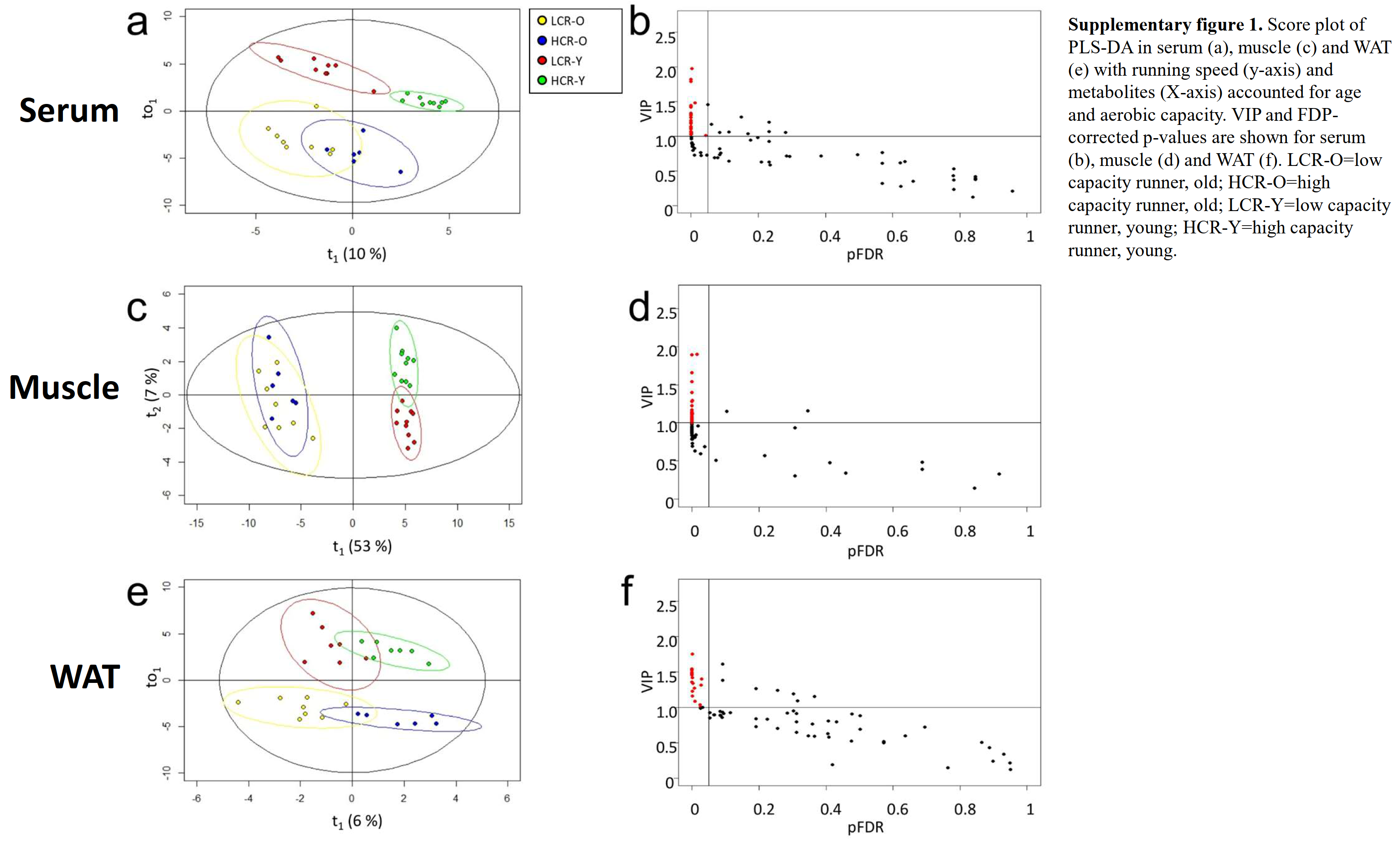

Supplement: Supplementary file 2 — High Resolution (TIF 1887 kb) [file 11357_2021_387_MOESM1_ESM.tif]
