# Supplementary material for: Interactive effects of aging and aerobic capacity on energy metabolism–related metabolites of serum, skeletal muscle, and white adipose tissue
Source: GeroScience. 2021 Jun 5;43(6):2679–91. doi: 10.1007/s11357-021-00387-1 (PMC8602622; doi:10.1007/s11357-021-00387-1)
Supplement: Supplementary file 3 — (DOCX 14 kb) [file 11357_2021_387_MOESM2_ESM.docx]

**Supplementary Table 1.** The R^2^Y and Q^2^ of PLS-DA model for aerobic capacity and aging effects.

| Tissue | R^2^_X_ | R^2^_Y_ | Q^2^ | p(R^2^_Y_) | p(Q^2^) |
| --- | --- | --- | --- | --- | --- |
| Serum | 0.397 | 0.568 | 0.443 | 0.005 | 0.005 |
| Muscle | 0.599 | 0.523 | 0.350 | 0.005 | 0.005 |
| WAT | 0.339 | 0.546 | 0.325 | 0.005 | 0.005 |
