# Supplementary material for: Interactive effects of aging and aerobic capacity on energy metabolism–related metabolites of serum, skeletal muscle, and white adipose tissue
Source: GeroScience. 2021 Jun 5;43(6):2679–91. doi: 10.1007/s11357-021-00387-1 (PMC8602622; doi:10.1007/s11357-021-00387-1)
Supplement: Supplementary file 4 — (DOCX 40 kb) [file 11357_2021_387_MOESM3_ESM.docx]

**Supplementary Table 2**. The univariate and multivariate analysis of Age, Aerobic Capacity (ACap), and their interaction on metabolites in serum, muscle and WAT

| **Serum**  **Metabolite** | **Univariate analysis** | | | | | | | | **Multivariate analysis** | | |
| --- | --- | --- | --- | --- | --- | --- | --- | --- | --- | --- | --- |
|  | **Uncorrected p-value** | | | | **pFDR** | **Regression coefficients** | | | **PCA loadings** | | **VIP** |
|  | **Age** | **ACap** | **Age×Acap** | **Model** |  | **Age** | **ACap** | **Age×Acap** | **1** | **2** |  |
| Lysine | 0.140 | 0.603 | 0.608 | 0.170 | 0.232 | 0.772 | -0.274 | -0.358 | 0.04 | 0.04 | 0.92 |
| Alanine | 0.001 | 0.723 | 0.572 | <0.001 | <0.001 | 1.336 | -0.134 | 0.284 | 0.14 | -0.05 | 1.02 |
| Glutamine | <0.001 | 0.909 | 0.746 | <0.001 | <0.001 | -1.844 | -0.028 | 0.107 | -0.18 | -0.03 | 1.23 |
| Betaine | 0.981 | 0.092 | 0.401 | 0.232 | 0.293 | -0.012 | -0.918 | 0.596 | 0.05 | 0.12 | 0.71 |
| Creatine | 0.001 | 0.852 | 0.831 | <0.001 | <0.001 | 1.376 | -0.074 | 0.112 | 0.15 | 0.04 | 0.98 |
| Taurine | 0.082 | 0.110 | 0.286 | 0.002 | 0.006 | -0.778 | 0.730 | -0.638 | -0.13 | -0.06 | 0.88 |
| Glyceraldehyde | <0.001 | 0.355 | 0.903 | <0.001 | <0.001 | 1.481 | 0.362 | -0.063 | 0.15 | -0.07 | 1.06 |
| Serine | 0.375 | 0.864 | 0.446 | 0.737 | 0.782 | 0.489 | 0.096 | -0.571 | 0.01 | 0.04 | 0.53 |
| Threonine | 0.171 | 0.381 | 0.419 | 0.544 | 0.623 | -0.751 | -0.487 | 0.596 | -0.06 | 0.05 | 0.28 |
| Allantoin | 0.288 | 0.648 | 0.728 | 0.403 | 0.495 | 0.571 | -0.249 | -0.251 | 0.04 | 0.02 | 0.73 |
| Hippuric acid | <0.001 | 0.945 | 0.224 | <0.001 | <0.001 | 1.648 | 0.015 | 0.348 | 0.18 | -0.03 | 1.30 |
| Leucine | <0.001 | 0.915 | 0.006 | <0.001 | <0.001 | 1.430 | -0.020 | 0.732 | 0.17 | -0.13 | 1.44 |
| Chenodeoxycholic acid | 0.238 | 0.844 | 0.373 | 0.563 | 0.637 | 0.645 | 0.109 | -0.659 | 0.01 | 0.17 | 0.63 |
| Valine | 0.211 | 0.974 | 0.003 | <0.001 | <0.001 | 0.424 | -0.011 | 1.458 | 0.12 | -0.26 | 1.79 |
| L-glutamic acid | 0.001 | 0.419 | 0.993 | <0.001 | <0.001 | 1.458 | 0.312 | 0.005 | 0.14 | -0.08 | 1.07 |
| Glycine | 0.047 | 0.165 | 0.553 | 0.006 | 0.012 | 0.927 | -0.650 | -0.364 | 0.07 | 0.16 | 1.48 |
| Citrulline | 0.001 | 0.814 | 0.331 | <0.001 | <0.001 | 1.282 | -0.085 | 0.466 | 0.15 | -0.05 | 1.11 |
| Proline | 0.001 | 0.219 | 0.168 | 0.003 | 0.006 | -1.622 | -0.558 | 0.833 | -0.12 | -0.02 | 0.80 |
| Choline | 0.059 | 0.288 | 0.165 | 0.001 | 0.002 | -0.818 | 0.461 | -0.805 | -0.13 | -0.01 | 0.90 |
| Ornithine | <0.001 | 0.899 | 0.453 | <0.001 | <0.001 | -1.430 | 0.042 | -0.333 | -0.16 | 0.04 | 1.13 |
| Hydroxyproline | <0.001 | 0.972 | 0.991 | <0.001 | <0.001 | 1.929 | 0.005 | 0.002 | 0.18 | -0.01 | 1.32 |
| Histidine | 0.115 | 0.235 | 0.131 | 0.002 | 0.005 | 0.694 | -0.531 | 0.904 | 0.11 | -0.08 | 0.85 |
| Isoleucine | 0.079 | 0.395 | 0.281 | 0.027 | 0.050 | -0.861 | 0.418 | 0.705 | -0.06 | -0.28 | 1.46 |
| Tryptophan | 0.116 | 0.806 | 0.170 | 0.097 | 0.149 | -0.804 | 0.126 | 0.951 | -0.04 | -0.26 | 1.28 |
| Myoinositol | <0.001 | 0.318 | 0.609 | <0.001 | <0.001 | 1.471 | -0.332 | 0.225 | 0.15 | 0.03 | 1.14 |
| Tyrosine | 0.519 | 0.875 | 0.827 | 0.831 | 0.846 | 0.357 | -0.089 | -0.163 | 0.01 | -0.02 | 0.38 |
| Phenylalanine | 0.102 | 0.203 | 0.865 | 0.023 | 0.044 | -0.794 | 0.627 | -0.109 | -0.10 | -0.20 | 1.01 |
| Asparagine | 0.594 | 0.928 | 0.809 | 0.730 | 0.782 | 0.293 | 0.050 | 0.180 | 0.03 | 0.03 | 0.37 |
| Carnitine | 0.058 | 0.550 | 0.971 | 0.051 | 0.085 | 0.958 | 0.300 | -0.024 | 0.09 | -0.15 | 0.73 |
| Acetoacetic acid | <0.001 | 0.922 | 0.173 | <0.001 | <0.001 | 1.960 | -0.026 | -0.484 | 0.16 | 0.10 | 1.33 |
| Creatinine | <0.001 | 0.555 | 1.000 | <0.001 | <0.001 | 1.518 | 0.219 | 0.000 | 0.15 | -0.10 | 1.06 |
| Succinate | 0.556 | 0.058 | 0.034 | 0.004 | 0.010 | -0.263 | 0.892 | -1.338 | -0.10 | 0.00 | 0.73 |
| L-methionine | 0.124 | 0.342 | 0.243 | 0.483 | 0.569 | -0.843 | -0.526 | 0.861 | -0.04 | -0.02 | 0.32 |
| deoxycytidine | 0.155 | 0.613 | 0.242 | 0.469 | 0.569 | -0.776 | -0.278 | 0.863 | -0.04 | -0.20 | 0.61 |
| Arginine | 0.191 | 0.280 | 0.029 | 0.120 | 0.178 | -0.671 | -0.565 | 1.562 | 0.02 | -0.21 | 0.95 |
| Aspartate | 0.003 | 0.572 | 0.741 | 0.002 | 0.004 | 1.374 | 0.249 | -0.193 | 0.13 | -0.05 | 0.86 |
| Homocysteine | 0.058 | 0.836 | 0.561 | 0.146 | 0.210 | 0.998 | 0.108 | -0.404 | 0.06 | 0.03 | 0.63 |
| Acetylcarnitine | 0.669 | 0.522 | 0.779 | 0.482 | 0.569 | -0.230 | -0.353 | -0.205 | -0.04 | 0.03 | 0.76 |
| Uracil | <0.001 | 0.239 | 0.040 | <0.001 | <0.001 | 2.059 | 0.382 | -0.908 | 0.15 | 0.08 | 1.18 |
| Niacinamide | 0.002 | 0.571 | 0.616 | 0.001 | 0.003 | 1.442 | 0.248 | -0.291 | 0.12 | -0.01 | 0.87 |
| D-glucuronic acid | 0.035 | 0.842 | 0.633 | 0.050 | 0.085 | 1.069 | -0.100 | -0.317 | 0.08 | 0.10 | 0.82 |
| D-ribose-5-phosphate | <0.001 | 0.883 | 0.437 | <0.001 | 0.001 | 1.587 | 0.060 | -0.422 | 0.12 | 0.10 | 1.03 |
| Sorbitol | 0.814 | 0.969 | 0.203 | 0.112 | 0.170 | -0.119 | 0.020 | -0.886 | -0.07 | 0.08 | 1.04 |
| Phosphoethanolamine | 0.011 | 0.009 | 0.291 | <0.001 | <0.001 | 0.971 | -1.029 | 0.522 | 0.13 | 0.11 | 1.31 |
| DimethylGlycine | <0.001 | 0.504 | 0.631 | <0.001 | <0.001 | -1.474 | 0.226 | -0.215 | -0.16 | -0.03 | 1.10 |
| 2-aminoisobutyric acid | 0.008 | 0.309 | 0.467 | 0.015 | 0.030 | 1.318 | 0.490 | -0.463 | 0.09 | -0.01 | 0.72 |
| Spermidine | 0.053 | 0.655 | 0.850 | 0.072 | 0.113 | -0.990 | -0.227 | 0.127 | -0.10 | 0.02 | 0.64 |
| Pantothenic acid | 0.013 | 0.543 | 0.230 | 0.055 | 0.089 | 1.286 | 0.306 | -0.810 | 0.07 | 0.05 | 0.76 |
| Aminodipic acid | <0.001 | 0.403 | 0.466 | <0.001 | <0.001 | 1.898 | 0.223 | -0.257 | 0.16 | 0.00 | 1.19 |
| Carnosine | <0.001 | 0.458 | 0.126 | <0.001 | <0.001 | 1.706 | -0.125 | 0.347 | 0.18 | -0.03 | 1.31 |
| Asymmetric dimethylarginine | 0.028 | 0.861 | 0.750 | 0.039 | 0.070 | 1.106 | 0.087 | -0.209 | 0.09 | -0.08 | 0.69 |
| Guanidinoacetic acid | 0.641 | 0.147 | 0.397 | 0.173 | 0.232 | -0.240 | 0.776 | -0.595 | -0.06 | -0.18 | 0.63 |
| Taurocholic acid | <0.001 | 0.915 | 0.377 | <0.001 | <0.001 | 1.848 | 0.032 | -0.358 | 0.16 | 0.08 | 1.21 |
| L-kynurenine | 0.023 | 0.562 | 0.349 | 0.001 | 0.002 | 0.996 | -0.250 | 0.539 | 0.12 | -0.06 | 0.91 |
| Trimethylamine-N-oxide | 0.046 | 0.693 | 0.129 | 0.137 | 0.199 | -1.049 | -0.206 | 1.072 | -0.04 | -0.17 | 0.98 |
| Symmetric dimethylarginine | 0.878 | 0.397 | 0.717 | 0.708 | 0.781 | -0.084 | 0.477 | -0.270 | -0.03 | -0.14 | 0.43 |
| 2-deoxyuridine | 0.026 | 0.829 | 0.653 | 0.045 | 0.079 | -1.128 | -0.108 | 0.297 | -0.09 | -0.11 | 0.69 |
| Sucrose | 0.446 | 0.390 | 0.336 | 0.800 | 0.839 | -0.421 | -0.488 | 0.726 | 0.02 | -0.01 | 0.12 |
| Xanthosine | 0.253 | 0.219 | 0.702 | 0.310 | 0.386 | 0.608 | 0.671 | -0.273 | 0.06 | -0.10 | 0.72 |
| IMP | 0.008 | 0.410 | 0.330 | <0.001 | <0.001 | 1.105 | -0.333 | 0.524 | 0.14 | -0.01 | 0.96 |
| Propionylcarnitine | 0.525 | 0.285 | 0.638 | 0.168 | 0.232 | -0.326 | 0.567 | 0.329 | -0.02 | -0.27 | 1.20 |
| 5-hydroxyindole-3-acetic acid | <0.001 | 0.925 | 0.610 | <0.001 | <0.001 | 1.460 | 0.033 | 0.234 | 0.16 | -0.02 | 1.11 |
| Glycocholic acid | 0.166 | 0.249 | 0.727 | 0.222 | 0.285 | -0.729 | -0.620 | 0.246 | -0.07 | 0.13 | 0.72 |
| Isobutyrylcarnitine | 0.131 | 0.718 | 0.006 | <0.001 | <0.001 | 0.512 | 0.123 | 1.320 | 0.11 | -0.26 | 1.82 |
| Taurochenodeoxycholic acid | 0.510 | 0.990 | 0.220 | 0.051 | 0.085 | -0.323 | -0.006 | -0.826 | -0.09 | 0.17 | 1.06 |
| Cystathionine | 0.431 | 0.925 | 0.854 | 0.592 | 0.661 | -0.429 | 0.052 | -0.135 | -0.05 | -0.04 | 0.35 |
| Cytidine | 0.020 | 0.462 | 0.728 | 0.024 | 0.047 | -1.158 | -0.360 | 0.225 | -0.10 | -0.03 | 0.73 |
| Cholic acid | 0.265 | 0.530 | 0.478 | 0.723 | 0.782 | -0.617 | -0.353 | 0.530 | -0.04 | 0.06 | 0.23 |
| GABA | 0.036 | 0.427 | 0.549 | 0.004 | 0.010 | -0.973 | 0.364 | -0.363 | -0.12 | 0.00 | 0.83 |
| Gamma-glutamylcysteine | 0.471 | 0.963 | 0.607 | 0.836 | 0.846 | -0.399 | -0.026 | 0.387 | -0.03 | -0.15 | 0.42 |
| Octanoylcarnitine | 0.448 | 0.716 | 0.419 | 0.827 | 0.846 | 0.420 | 0.205 | -0.609 | 0.01 | -0.04 | 0.39 |
| Kynurenic acid | <0.001 | 0.407 | 0.272 | <0.001 | <0.001 | 1.693 | -0.286 | -0.505 | 0.14 | 0.15 | 1.43 |
| Isovalerylcarnitine | 0.697 | 0.014 | 0.565 | 0.001 | 0.002 | 0.162 | 1.108 | 0.326 | 0.03 | -0.36 | 1.97 |
| AMP | 0.613 | 0.737 | 0.712 | 0.536 | 0.623 | -0.273 | -0.186 | -0.270 | -0.03 | 0.01 | 0.62 |
| Decanoylcarnitine | 0.998 | 0.653 | 0.833 | 0.957 | 0.957 | 0.002 | 0.257 | -0.160 | -0.01 | -0.14 | 0.21 |
| 4-pyridoxic acid | <0.001 | 0.880 | 0.892 | <0.001 | <0.001 | 1.549 | 0.056 | -0.067 | 0.15 | -0.04 | 1.03 |
| Folic acid | <0.001 | 0.998 | 0.791 | <0.001 | <0.001 | 1.823 | 0.001 | 0.071 | 0.18 | 0.01 | 1.27 |
| Inosine | 0.287 | 0.032 | 0.161 | 0.178 | 0.235 | 0.552 | 1.178 | -0.995 | -0.01 | -0.08 | 0.59 |
| NAD | 0.003 | 0.540 | 0.218 | <0.001 | <0.001 | 1.155 | -0.228 | 0.615 | 0.15 | -0.02 | 1.07 |
| Nicotinic acid | 0.626 | 0.299 | 0.767 | 0.216 | 0.282 | 0.253 | 0.556 | 0.209 | 0.04 | -0.16 | 1.06 |
| Adenosine | 0.062 | 0.466 | 0.612 | 0.014 | 0.029 | -0.897 | 0.348 | -0.321 | -0.12 | -0.01 | 0.77 |
| Hexanoylcarnitine | 0.651 | 0.023 | 0.358 | 0.072 | 0.113 | 0.224 | 1.207 | -0.624 | -0.02 | -0.20 | 1.06 |
| Xanthine | <0.001 | 0.014 | 0.742 | <0.001 | <0.001 | 1.812 | 0.459 | 0.078 | 0.18 | -0.09 | 1.42 |
| 1-methylhistamine | 0.223 | 0.373 | 0.558 | 0.033 | 0.060 | -0.592 | -0.442 | -0.384 | -0.08 | 0.13 | 1.17 |
| Cytosine | 0.474 | 0.217 | 0.927 | 0.172 | 0.232 | -0.368 | 0.658 | 0.064 | -0.05 | -0.19 | 1.07 |
| Adenine | <0.001 | 0.933 | 0.105 | <0.001 | <0.001 | 1.866 | 0.029 | -0.761 | 0.15 | 0.11 | 1.32 |

Note. Gray shading indicated VIP ≥ 1 and pFDR < 0.05.

| **Muscle**  **Metabolite** | **Univariate analysis** | | | | | | | | **Multivariate analysis** | | |
| --- | --- | --- | --- | --- | --- | --- | --- | --- | --- | --- | --- |
|  | **Uncorrected p-value** | | | | **pFDR** | **Regression coefficients** | | | **PCA loadings** | | **VIP** |
|  | **Age** | **ACap** | **Age×Acap** | **Model** |  | **Age** | **ACap** | **Age×Acap** | **1** | **2** |  |
| Alanine | 0.000 | 0.834 | 0.052 | 0.000 | <0.001 | 2.048 | -0.009 | -0.108 | 0.16 | 0.06 | 1.11 |
| Taurine | 0.000 | 0.697 | 0.031 | 0.000 | <0.001 | -2.117 | -0.044 | 0.331 | -0.15 | -0.10 | 1.15 |
| Hydroxyproline | 0.000 | 0.995 | 0.014 | 0.000 | <0.001 | 2.078 | 0.000 | -0.181 | 0.16 | 0.07 | 1.13 |
| Creatine | 0.246 | 0.705 | 0.201 | 0.015 | 0.018 | -0.539 | 0.182 | -0.809 | -0.09 | 0.25 | 0.96 |
| Glutamine | 0.000 | 0.520 | 0.254 | 0.000 | <0.001 | -2.044 | -0.104 | 0.242 | -0.16 | -0.08 | 1.07 |
| Acetoacetic acid | 0.000 | 0.889 | 0.007 | 0.000 | <0.001 | 2.156 | -0.024 | -0.651 | 0.15 | 0.20 | 1.40 |
| Glycine | 0.000 | 0.214 | 0.558 | 0.000 | <0.001 | -1.562 | 0.368 | -0.223 | -0.14 | -0.03 | 0.99 |
| Niacinamide | 0.001 | 0.158 | 0.085 | 0.000 | <0.001 | 1.153 | -0.482 | 0.771 | 0.12 | 0.05 | 0.90 |
| Carnitine | 0.548 | 0.714 | 0.521 | 0.917 | 0.917 | 0.326 | 0.208 | -0.473 | 0.00 | 0.19 | 0.33 |
| Serine | 0.000 | 0.015 | 0.525 | 0.000 | <0.001 | -1.628 | 0.587 | -0.191 | -0.14 | -0.18 | 1.23 |
| Threonine | 0.000 | 0.323 | 0.786 | 0.000 | <0.001 | -1.711 | -0.321 | 0.114 | -0.13 | -0.02 | 0.95 |
| Acetylcarnitine | 0.000 | 0.043 | 0.021 | 0.001 | 0.001 | -1.868 | -0.900 | 1.356 | -0.09 | -0.06 | 0.73 |
| L-glutamic acid | 0.000 | 0.713 | 0.553 | 0.000 | <0.001 | -1.905 | 0.084 | 0.177 | -0.15 | -0.09 | 1.08 |
| Choline | 0.005 | 0.994 | 0.574 | 0.000 | <0.001 | -1.204 | 0.003 | -0.304 | -0.11 | -0.07 | 0.83 |
| Glutathione | 0.000 | 0.219 | 0.347 | 0.000 | <0.001 | -1.717 | 0.255 | -0.252 | -0.16 | 0.03 | 1.03 |
| Creatinine | 0.000 | 0.985 | 0.765 | 0.000 | <0.001 | -1.836 | -0.005 | 0.100 | -0.15 | -0.08 | 0.99 |
| Proline | 0.000 | 0.278 | 0.202 | 0.000 | <0.001 | -1.972 | -0.319 | 0.489 | -0.14 | -0.07 | 0.95 |
| Hypoxanthine | 0.000 | 0.068 | 0.274 | 0.000 | <0.001 | -1.669 | -0.755 | 0.578 | -0.12 | 0.18 | 0.83 |
| Valine | 0.000 | 0.572 | 0.639 | 0.000 | <0.001 | -1.699 | 0.185 | 0.198 | -0.14 | -0.03 | 1.07 |
| Succinate | 0.000 | 0.004 | 0.010 | 0.000 | <0.001 | -1.191 | 0.807 | -0.914 | -0.14 | -0.02 | 0.95 |
| Leucine | 0.001 | 0.795 | 0.516 | 0.001 | 0.001 | -1.492 | -0.112 | 0.364 | -0.11 | 0.03 | 0.78 |
| Inosine | 0.923 | 0.228 | 0.276 | 0.011 | 0.014 | -0.044 | -0.581 | -0.680 | -0.04 | 0.45 | 1.90 |
| Glyceraldehyde | 0.014 | 0.391 | 0.906 | 0.007 | 0.009 | -1.157 | -0.403 | 0.072 | -0.10 | 0.21 | 0.81 |
| Allantoin | 0.000 | 0.690 | 0.149 | 0.000 | <0.001 | -1.287 | 0.129 | -0.616 | -0.14 | 0.11 | 1.07 |
| Betaine | 0.000 | 0.037 | 0.355 | 0.000 | <0.001 | -1.590 | -0.864 | 0.482 | -0.11 | 0.01 | 1.01 |
| Citrulline | 0.000 | 0.734 | 0.707 | 0.000 | <0.001 | -1.811 | 0.092 | 0.132 | -0.14 | -0.08 | 1.03 |
| Asparagine | 0.000 | 0.392 | 0.068 | 0.000 | <0.001 | -1.754 | 0.120 | -0.340 | -0.16 | 0.02 | 1.08 |
| Tyrosine | 0.001 | 0.733 | 0.748 | 0.000 | <0.001 | -1.400 | -0.130 | -0.159 | -0.13 | 0.15 | 0.90 |
| Aspartate | 0.000 | 0.721 | 0.959 | 0.000 | <0.001 | -1.552 | -0.128 | -0.024 | -0.13 | 0.05 | 0.88 |
| Isoleucine | 0.000 | 0.976 | 0.602 | 0.000 | <0.001 | -1.781 | 0.009 | 0.209 | -0.14 | -0.03 | 0.98 |
| Ornithine | 0.004 | 0.537 | 0.004 | 0.000 | <0.001 | -0.899 | 0.185 | -1.193 | -0.13 | 0.25 | 1.54 |
| Phenylalanine | 0.000 | 0.717 | 0.701 | 0.000 | <0.001 | -1.591 | 0.113 | -0.155 | -0.14 | 0.07 | 0.92 |
| L-methionine | 0.000 | 0.259 | 0.392 | 0.000 | <0.001 | -1.874 | -0.348 | 0.341 | -0.14 | 0.03 | 0.92 |
| Uracil | 0.804 | 0.755 | 0.365 | 0.316 | 0.345 | 0.128 | -0.168 | -0.638 | -0.02 | 0.23 | 1.16 |
| Tryptophan | 0.009 | 0.733 | 0.005 | 0.000 | <0.001 | -0.830 | 0.107 | -1.207 | -0.13 | 0.24 | 1.66 |
| Xanthine | 0.275 | 0.510 | 0.765 | 0.273 | 0.307 | 0.565 | -0.354 | -0.208 | 0.03 | 0.34 | 0.93 |
| IMP | 0.380 | 0.306 | 0.564 | 0.667 | 0.686 | -0.471 | -0.574 | 0.418 | -0.02 | 0.24 | 0.39 |
| Pantothenic acid | 0.000 | 0.036 | 0.230 | 0.000 | <0.001 | -1.526 | 0.545 | -0.394 | -0.14 | -0.02 | 1.05 |
| Chenodeoxycholic acid | 0.002 | 0.435 | 0.156 | 0.009 | 0.011 | 1.494 | 0.370 | -0.885 | 0.08 | 0.20 | 0.84 |
| Sorbitol | 0.000 | 0.074 | 0.386 | 0.000 | <0.001 | -1.470 | 0.537 | -0.332 | -0.14 | -0.02 | 1.03 |
| Hippuric acid | 0.000 | 0.095 | 0.039 | 0.000 | <0.001 | -1.279 | 0.478 | -0.778 | -0.14 | 0.02 | 0.97 |
| deoxycytidine | 0.071 | 0.856 | 0.743 | 0.032 | 0.038 | -0.876 | -0.090 | -0.210 | -0.09 | 0.09 | 0.68 |
| Taurocholic acid | 0.118 | 0.860 | 0.817 | 0.189 | 0.216 | 0.807 | -0.093 | -0.158 | 0.05 | 0.26 | 0.57 |
| 2-aminoisobutyric acid | 0.074 | 0.205 | 0.299 | 0.277 | 0.307 | 0.942 | 0.689 | -0.729 | 0.04 | -0.06 | 0.30 |
| Cytidine | 0.504 | 0.701 | 0.937 | 0.666 | 0.686 | -0.357 | -0.214 | -0.057 | -0.04 | 0.25 | 0.48 |
| Octanoylcarnitine | 0.000 | 0.239 | 0.247 | 0.001 | 0.001 | -1.632 | -0.513 | 0.654 | -0.10 | -0.02 | 0.69 |
| Phosphoethanolamine | 0.018 | 0.066 | 0.184 | 0.060 | 0.071 | -1.204 | -0.956 | 0.886 | -0.06 | 0.03 | 0.51 |
| Isobutyrylcarnitine | 0.006 | 0.878 | 0.749 | 0.001 | 0.001 | -1.210 | -0.067 | -0.179 | -0.11 | 0.09 | 0.78 |
| Folic acid | 0.000 | 0.401 | 0.011 | 0.000 | <0.001 | 2.171 | 0.118 | -0.489 | 0.15 | 0.15 | 1.17 |
| Hexanoylcarnitine | 0.125 | 0.527 | 0.444 | 0.433 | 0.459 | -0.818 | -0.346 | 0.545 | -0.04 | 0.00 | 0.34 |
| Decanoylcarnitine | 0.000 | 0.193 | 0.349 | 0.000 | <0.001 | -1.878 | -0.410 | 0.381 | -0.14 | 0.06 | 0.91 |
| Guanidinoacetic acid | 0.000 | 0.906 | 0.969 | 0.000 | <0.001 | -1.706 | 0.035 | 0.015 | -0.14 | -0.07 | 0.94 |
| DimethylGlycine | 0.009 | 0.321 | 0.148 | 0.000 | <0.001 | -1.031 | 0.386 | -0.738 | -0.12 | 0.06 | 0.86 |
| Sucrose | 0.000 | 0.818 | 0.050 | 0.000 | <0.001 | -1.216 | 0.069 | -0.785 | -0.13 | 0.09 | 1.28 |
| L-kynurenine | 0.438 | 0.410 | 0.522 | 0.831 | 0.843 | 0.420 | 0.467 | -0.470 | 0.01 | -0.02 | 0.14 |
| Trimethylamine-N-oxide | 0.012 | 0.295 | 0.510 | 0.020 | 0.025 | -1.240 | -0.514 | 0.418 | -0.08 | 0.03 | 0.59 |
| Propionylcarnitine | 0.000 | 0.790 | 0.844 | 0.000 | <0.001 | -1.496 | -0.106 | 0.101 | -0.12 | 0.06 | 0.79 |
| NAD | 0.000 | 0.510 | 0.097 | 0.000 | <0.001 | 1.804 | 0.263 | -0.875 | 0.11 | 0.23 | 1.08 |
| Isovalerylcarnitine | 0.729 | 0.002 | 0.130 | 0.001 | 0.002 | -0.146 | 1.487 | -0.883 | -0.06 | -0.09 | 1.29 |
| GABA | 0.000 | 0.333 | 0.615 | 0.000 | <0.001 | -1.550 | 0.301 | -0.202 | -0.14 | 0.01 | 0.95 |
| Spermidine | 0.000 | 0.528 | 0.007 | 0.000 | <0.001 | -1.379 | 0.134 | -0.791 | -0.15 | 0.16 | 1.28 |
| Glycocholic acid | 0.010 | 0.841 | 0.826 | 0.006 | 0.009 | 1.225 | 0.094 | -0.133 | 0.09 | 0.04 | 0.63 |
| 2-deoxyuridine | 0.027 | 0.750 | 0.364 | 0.001 | 0.001 | -0.953 | -0.138 | -0.513 | -0.10 | 0.18 | 1.12 |
| AMP | 0.000 | 0.540 | 0.872 | 0.000 | <0.001 | -1.716 | -0.172 | -0.059 | -0.14 | 0.10 | 1.00 |
| Adenosine | 0.000 | 0.929 | 0.836 | 0.000 | <0.001 | -1.879 | -0.020 | 0.059 | -0.15 | -0.06 | 1.02 |
| Adenine | 0.000 | 0.894 | 0.317 | 0.000 | <0.001 | -1.798 | 0.022 | -0.218 | -0.15 | 0.01 | 1.07 |
| Cytosine | 0.021 | 0.079 | 0.226 | 0.000 | <0.001 | -0.942 | 0.734 | -0.649 | -0.11 | -0.01 | 0.80 |
| Kynurenic acid | 0.150 | 0.877 | 0.620 | 0.382 | 0.411 | 0.762 | 0.084 | -0.351 | 0.05 | 0.09 | 0.48 |
| Taurochenodeoxycholic acid | 0.867 | 0.802 | 0.152 | 0.089 | 0.103 | -0.082 | 0.129 | -0.972 | -0.05 | 0.16 | 1.15 |
| Normetanephrine | 0.000 | 0.187 | 0.060 | 0.000 | <0.001 | -1.763 | 0.405 | 0.761 | -0.12 | -0.20 | 1.90 |
| 4-pyridoxic acid | 0.000 | 0.904 | 0.557 | 0.000 | <0.001 | -1.412 | -0.043 | -0.272 | -0.13 | 0.08 | 0.94 |

Note. Gray shading indicated VIP ≥ 1 and pFDR < 0.05.

| **WAT**  **Metabolite** | **Univariate analysis** | | | | | | | | **Multivariate analysis** | | |
| --- | --- | --- | --- | --- | --- | --- | --- | --- | --- | --- | --- |
|  | **Uncorrected p-value** | | | | **pFDR** | **Regression coefficients** | | | **PCA loadings** | | **VIP** |
|  | **Age** | **ACap** | **Age×Acap** | **Model** |  | **Age** | **ACap** | **Age×Acap** | **1** | **2** |  |
| Taurine | 0.005 | 0.956 | 0.264 | 0.000 | <0.001 | -1.169 | 0.020 | -0.586 | -0.19 | -0.01 | 1.16 |
| Alanine | 0.000 | 0.743 | 0.692 | 0.000 | <0.001 | 1.948 | 0.045 | -0.077 | 0.23 | 0.03 | 1.53 |
| Choline | 0.004 | 0.020 | 0.151 | 0.008 | 0.028 | -1.477 | -1.115 | 0.935 | -0.12 | 0.12 | 1.40 |
| Acetoacetic acid | 0.000 | 0.002 | 0.001 | 0.000 | <0.001 | 2.378 | 0.816 | -1.344 | 0.19 | -0.13 | 1.50 |
| Myoinositol | 0.060 | 0.372 | 0.735 | 0.096 | 0.190 | 1.024 | 0.458 | -0.242 | 0.08 | -0.16 | 0.84 |
| Glutathione | 0.542 | 0.223 | 0.147 | 0.054 | 0.115 | -0.312 | 0.613 | -1.034 | -0.10 | -0.19 | 0.93 |
| Hypoxanthine | 0.088 | 0.899 | 0.563 | 0.233 | 0.347 | -0.962 | -0.067 | 0.433 | -0.08 | -0.12 | 0.60 |
| Creatine | 0.570 | 0.472 | 0.790 | 0.564 | 0.637 | 0.326 | -0.402 | 0.208 | 0.08 | 0.12 | 0.60 |
| Hydroxyproline | 0.000 | 0.221 | 0.369 | 0.000 | <0.001 | 2.006 | 0.132 | -0.136 | 0.23 | 0.01 | 1.54 |
| Glutamine | 0.438 | 0.236 | 0.592 | 0.198 | 0.312 | -0.423 | 0.633 | -0.398 | -0.09 | -0.15 | 0.92 |
| Glycine | 0.184 | 0.228 | 0.881 | 0.162 | 0.284 | -0.726 | -0.638 | 0.110 | -0.10 | 0.07 | 0.92 |
| L-glutamic acid | 0.022 | 0.952 | 0.848 | 0.022 | 0.065 | -1.186 | -0.029 | 0.129 | -0.16 | 0.10 | 0.90 |
| Serine | 0.275 | 0.118 | 0.141 | 0.423 | 0.502 | 0.622 | 0.877 | -1.160 | 0.00 | -0.24 | 0.88 |
| Succinate | 0.177 | 0.727 | 0.775 | 0.295 | 0.405 | 0.760 | -0.188 | -0.216 | 0.06 | 0.14 | 0.63 |
| Threonine | 0.074 | 0.452 | 0.890 | 0.040 | 0.091 | -0.930 | 0.370 | -0.095 | -0.15 | -0.10 | 0.93 |
| Inosine | 0.955 | 0.072 | 0.458 | 0.180 | 0.303 | -0.030 | 0.977 | -0.550 | -0.03 | -0.29 | 1.19 |
| Ornithine | 0.126 | 0.249 | 0.515 | 0.303 | 0.407 | 0.867 | 0.628 | -0.494 | 0.07 | -0.24 | 0.81 |
| Niacinamide | 0.116 | 0.957 | 0.375 | 0.016 | 0.053 | -0.781 | 0.025 | -0.590 | -0.14 | -0.11 | 0.85 |
| Chenodeoxycholic acid | 0.000 | 0.685 | 0.450 | 0.000 | <0.001 | 1.955 | 0.080 | -0.210 | 0.22 | -0.03 | 1.48 |
| Proline | 0.165 | 0.614 | 0.549 | 0.495 | 0.572 | -0.804 | -0.278 | 0.466 | -0.11 | 0.07 | 0.52 |
| Leucine | 0.008 | 0.670 | 0.654 | 0.005 | 0.025 | 1.325 | -0.191 | -0.282 | 0.11 | 0.04 | 1.04 |
| Valine | 0.004 | 0.536 | 0.842 | 0.002 | 0.009 | -1.396 | -0.264 | 0.119 | -0.18 | 0.04 | 1.09 |
| Uracil | 0.110 | 0.942 | 0.657 | 0.261 | 0.365 | -0.903 | -0.039 | 0.334 | -0.11 | -0.06 | 0.59 |
| Arginine | 0.665 | 0.856 | 0.981 | 0.935 | 0.948 | -0.257 | -0.105 | 0.019 | -0.01 | -0.13 | 0.22 |
| Adenosine | 0.006 | 0.564 | 0.632 | 0.006 | 0.025 | 1.395 | 0.260 | -0.304 | 0.17 | -0.03 | 1.01 |
| Betaine | 0.090 | 0.029 | 0.465 | 0.040 | 0.091 | -0.879 | -1.124 | 0.505 | -0.10 | 0.26 | 1.38 |
| Aspartate | 0.998 | 0.369 | 0.726 | 0.417 | 0.502 | -0.002 | -0.495 | -0.269 | -0.04 | 0.07 | 0.69 |
| Tyrosine | 0.350 | 0.897 | 0.765 | 0.381 | 0.476 | -0.529 | -0.071 | -0.229 | -0.12 | 0.01 | 0.52 |
| Xanthine | 0.170 | 0.965 | 0.887 | 0.201 | 0.312 | -0.759 | 0.023 | -0.105 | -0.10 | -0.11 | 0.65 |
| Asparagine | 0.004 | 0.120 | 0.088 | 0.000 | <0.001 | -1.083 | 0.537 | -0.834 | -0.20 | -0.12 | 1.36 |
| Hippuric acid | 0.047 | 0.987 | 0.596 | 0.098 | 0.190 | -1.084 | 0.008 | 0.381 | -0.14 | 0.03 | 0.73 |
| Carnitine | 0.014 | 0.433 | 0.390 | 0.009 | 0.031 | -1.238 | 0.361 | 0.556 | -0.13 | -0.01 | 1.00 |
| Acetylcarnitine | 0.652 | 0.786 | 0.583 | 0.950 | 0.950 | -0.268 | -0.156 | 0.445 | -0.01 | 0.09 | 0.12 |
| Pantothenic acid | 0.055 | 0.146 | 0.074 | 0.247 | 0.360 | 1.093 | 0.790 | -1.381 | 0.03 | -0.18 | 0.77 |
| Phenylalanine | 0.112 | 0.607 | 0.432 | 0.023 | 0.067 | -0.802 | 0.246 | -0.530 | -0.15 | -0.07 | 0.90 |
| Tryptophan | 0.178 | 0.304 | 0.145 | 0.498 | 0.572 | 0.780 | 0.573 | -1.156 | 0.00 | -0.16 | 0.50 |
| L-methionine | 0.053 | 0.717 | 0.860 | 0.031 | 0.084 | -1.003 | 0.175 | -0.120 | -0.16 | -0.07 | 0.89 |
| Citrulline | 0.555 | 0.001 | 0.009 | 0.000 | 0.001 | -0.235 | 1.401 | -1.510 | -0.14 | -0.18 | 1.75 |
| Sorbitol | 0.117 | 0.139 | 0.086 | 0.339 | 0.431 | -0.897 | -0.818 | 1.349 | -0.04 | 0.09 | 0.80 |
| Allantoin | 0.028 | 0.586 | 0.824 | 0.033 | 0.085 | -1.153 | -0.264 | 0.151 | -0.15 | 0.04 | 0.89 |
| Creatinine | 0.007 | 0.199 | 0.125 | 0.043 | 0.095 | -1.478 | -0.640 | 1.086 | -0.11 | 0.14 | 0.91 |
| Deoxycytidine | 0.000 | 0.022 | 0.217 | 0.000 | 0.001 | 1.711 | 0.930 | -0.678 | 0.14 | -0.19 | 1.42 |
| Guanosine | 0.908 | 0.307 | 0.873 | 0.625 | 0.695 | 0.066 | 0.577 | -0.125 | 0.02 | -0.17 | 0.72 |
| Isobutyrylcarnitine | 0.000 | 0.747 | 0.506 | 0.000 | <0.001 | -1.652 | 0.118 | 0.343 | -0.20 | -0.03 | 1.23 |
| Xanthosine | 0.150 | 0.122 | 0.208 | 0.389 | 0.477 | 0.825 | 0.864 | -0.981 | 0.02 | -0.19 | 0.91 |
| Phosphoethanolamine | 0.005 | 0.045 | 0.291 | 0.007 | 0.027 | -1.422 | -0.948 | 0.679 | -0.15 | 0.22 | 1.32 |
| NAD | 0.000 | 0.594 | 0.405 | 0.000 | <0.001 | 1.965 | 0.107 | -0.235 | 0.22 | -0.03 | 1.47 |
| GABA | 0.148 | 0.020 | 0.026 | 0.096 | 0.190 | 0.775 | 1.251 | -1.675 | -0.02 | -0.23 | 1.27 |
| Sucrose | 0.589 | 0.535 | 0.469 | 0.903 | 0.930 | -0.319 | -0.356 | 0.587 | 0.00 | -0.07 | 0.34 |
| AMP | 0.187 | 0.040 | 0.031 | 0.000 | 0.002 | -0.553 | 0.860 | -1.276 | -0.16 | -0.18 | 1.34 |
| Dimethylglycine | 0.077 | 0.026 | 0.057 | 0.139 | 0.255 | 0.974 | 1.215 | -1.440 | 0.00 | -0.24 | 1.24 |
| Guanidinoacetic acid | 0.111 | 0.858 | 0.991 | 0.142 | 0.255 | -0.874 | -0.093 | -0.009 | -0.14 | 0.08 | 0.70 |
| Cytidine | 0.561 | 0.546 | 0.843 | 0.836 | 0.887 | 0.342 | 0.345 | -0.158 | 0.02 | 0.00 | 0.43 |
| L-kynurenine | 0.371 | 0.224 | 0.945 | 0.182 | 0.303 | 0.487 | -0.647 | 0.051 | 0.06 | 0.04 | 0.95 |
| Spermidine | 0.919 | 0.435 | 0.518 | 0.803 | 0.865 | 0.060 | 0.446 | -0.518 | -0.03 | -0.23 | 0.51 |
| Taurocholic acid | 0.071 | 0.497 | 0.761 | 0.031 | 0.084 | -0.929 | 0.330 | -0.207 | -0.14 | -0.02 | 0.94 |
| Propionylcarnitine | 0.006 | 0.950 | 0.603 | 0.006 | 0.025 | -1.381 | 0.028 | 0.329 | -0.16 | -0.08 | 0.99 |
| Taurochenodeoxycholic acid | 0.605 | 0.313 | 0.507 | 0.193 | 0.312 | 0.281 | -0.536 | -0.493 | -0.02 | 0.16 | 0.80 |
| 2-aminoisobutyric acid | 0.193 | 0.396 | 0.546 | 0.036 | 0.090 | 0.664 | 0.416 | 0.415 | 0.08 | -0.12 | 0.86 |
| Glycocholic acid | 0.756 | 0.081 | 0.523 | 0.257 | 0.365 | -0.171 | -0.961 | 0.481 | -0.02 | 0.26 | 1.16 |
| Isovalerylcarnitine | 0.012 | 0.138 | 0.861 | 0.001 | 0.008 | -1.162 | 0.639 | 0.104 | -0.16 | -0.09 | 1.27 |
| Trimethylamine-N-oxide | 0.364 | 0.721 | 0.310 | 0.698 | 0.764 | -0.531 | -0.201 | 0.813 | -0.03 | -0.03 | 0.15 |
| Octanoylcarnitine | 0.058 | 0.005 | 0.056 | 0.038 | 0.091 | -0.990 | -1.481 | 1.364 | -0.03 | 0.32 | 1.61 |
| Decanoylcarnitine | 0.000 | 0.165 | 0.213 | 0.000 | <0.001 | -2.019 | -0.317 | 0.399 | -0.21 | 0.01 | 1.46 |
| Hexanoylcarnitine | 0.407 | 0.764 | 0.568 | 0.860 | 0.898 | -0.490 | -0.171 | 0.460 | -0.05 | 0.11 | 0.24 |
| Cytosine | 0.080 | 0.652 | 0.481 | 0.017 | 0.053 | -0.875 | 0.212 | -0.468 | -0.14 | -0.05 | 0.92 |
| 4-pyridoxic acid | 0.157 | 0.710 | 0.141 | 0.323 | 0.419 | 0.803 | 0.201 | -1.144 | -0.01 | -0.01 | 0.19 |
| Kynurenic acid | 0.710 | 0.102 | 0.833 | 0.207 | 0.314 | -0.202 | -0.887 | 0.157 | -0.02 | 0.08 | 1.09 |
| Adenine | 0.339 | 0.797 | 0.669 | 0.309 | 0.407 | -0.535 | 0.138 | -0.324 | -0.09 | 0.01 | 0.58 |
| Normetanephrine | 0.021 | 0.237 | 0.177 | 0.118 | 0.224 | -1.293 | -0.616 | 0.996 | -0.12 | 0.13 | 0.83 |

Note. Gray shading indicated VIP ≥ 1 and pFDR < 0.05.
