# Supplementary material for: Interactive effects of aging and aerobic capacity on energy metabolism–related metabolites of serum, skeletal muscle, and white adipose tissue
Source: GeroScience. 2021 Jun 5;43(6):2679–91. doi: 10.1007/s11357-021-00387-1 (PMC8602622; doi:10.1007/s11357-021-00387-1)
Supplement: Supplementary file 5 — (DOCX 23 kb) [file 11357_2021_387_MOESM4_ESM.docx]

**Supplementary Table 3.** Pathway analysis of the metabolites significantly differing by aerobic capacity, age and aerobic capacity*age in serum, muscle and WAT.

| **Factor** | **Tissue** | **Pathway** | **p** | **FDR** | **Class of significant findings** |
| --- | --- | --- | --- | --- | --- |
| Aerobic capacity | Serum | Purine metabolism | 0.044 | 1.000 | Nucleotide metabolism |
|  | Muscle | - | - | - |  |
|  | WAT | Glycine, serine and threonine metabolism | 0.001 | 0.122 | Amino acid metabolism |
|  |  |  |  |  |  |
| Age | Serum | Arginine biosynthesis | 0.000 | 0.017 | Amino acid metabolism |
|  |  | Aminoacyl-tRNA biosynthesis | 0.001 | 0.025 | tRNA metabolism |
|  |  | Arginine and proline metabolism | 0.001 | 0.040 | Amino acid metabolism |
|  |  | Alanine, aspartate and glutamate metabolism | 0.003 | 0.070 | Amino acid metabolism |
|  |  | Nicotinate and nicotinamide metabolism | 0.004 | 0.072 | Metabolism of cofactors and vitamins |
|  |  | Ascorbate and aldarate metabolism | 0.021 | 0.293 | Metabolism of cofactors and vitamins |
|  |  | Butanoate metabolism | 0.045 | 0.537 | Lipid metabolism |
|  |  | Histidine metabolism | 0.051 | 0.537 | Amino acid metabolism |
|  |  | Purine metabolism | 0.063 | 0.587 |  |
|  |  | Pantothenate and CoA biosynthesis | 0.070 | 0.587 |  |
|  |  | beta-Alanine metabolism | 0.083 | 0.636 |  |
|  |  | Synthesis and degradation of ketone bodies | 0.111 | 0.758 |  |
|  |  | D-Glutamine and D-glutamate metabolism | 0.132 | 0.758 |  |
|  |  | Nitrogen metabolism | 0.132 | 0.758 |  |
|  |  | Glutathione metabolism | 0.136 | 0.758 |  |
|  |  | Inositol phosphate metabolism | 0.152 | 0.758 |  |
|  |  | Glyoxylate and dicarboxylate metabolism | 0.168 | 0.758 |  |
|  |  | Valine, leucine and isoleucine biosynthesis | 0.172 | 0.758 |  |
|  |  | Taurine and hypotaurine metabolism | 0.172 | 0.758 |  |
|  |  | Glycine, serine and threonine metabolism | 0.185 | 0.763 |  |
|  |  | One carbon pool by folate | 0.191 | 0.763 |  |
|  |  | Pyrimidine metabolism | 0.228 | 0.827 |  |
|  |  | Valine, leucine and isoleucine degradation | 0.237 | 0.827 |  |
|  |  | Tryptophan metabolism | 0.246 | 0.827 |  |
|  |  | Phenylalanine metabolism | 0.246 | 0.827 |  |
|  |  | Primary bile acid biosynthesis | 0.289 | 0.935 |  |
|  |  | Pentose and glucuronate interconversions | 0.346 | 1.000 |  |
|  |  | Selenocompound metabolism | 0.376 | 1.000 |  |
|  |  | Sphingolipid metabolism | 0.391 | 1.000 |  |
|  |  | Pentose phosphate pathway | 0.391 | 1.000 |  |
|  |  | Galactose metabolism | 0.472 | 1.000 |  |
|  |  | Folate biosynthesis | 0.472 | 1.000 |  |
|  |  | Phosphatidylinositol signaling system | 0.485 | 1.000 |  |
|  |  | Porphyrin and chlorophyll metabolism | 0.509 | 1.000 |  |
|  |  | Glycerophospholipid metabolism | 0.575 | 1.000 |  |
|  |  | Tyrosine metabolism | 0.632 | 1.000 |  |
|  | Muscle | Aminoacyl-tRNA biosynthesis | 0.000 | 0.000 | tRNA metabolism |
|  |  | Valine, leucine and isoleucine biosynthesis | 0.000 | 0.001 | Amino acid metabolism |
|  |  | Glycine, serine and threonine metabolism | 0.000 | 0.003 | Amino acid metabolism |
|  |  | Arginine biosynthesis | 0.000 | 0.005 | Amino acid metabolism |
|  |  | Alanine, aspartate and glutamate metabolism | 0.000 | 0.007 | Amino acid metabolism |
|  |  | Arginine and proline metabolism | 0.002 | 0.026 | Amino acid metabolism |
|  |  | Phenylalanine, tyrosine and tryptophan biosynthesis | 0.003 | 0.041 | Amino acid metabolism |
|  |  | Nicotinate and nicotinamide metabolism | 0.005 | 0.053 | Metabolism of cofactors and vitamins |
|  |  | Nitrogen metabolism | 0.008 | 0.069 | Nitrogen metabolism |
|  |  | D-Glutamine and D-glutamate metabolism | 0.008 | 0.069 | Amino acid metabolism |
|  |  | Glutathione metabolism | 0.029 | 0.223 | Amino acid metabolism |
|  |  | Phenylalanine metabolism | 0.033 | 0.232 | Amino acid metabolism |
|  |  | Glyoxylate and dicarboxylate metabolism | 0.041 | 0.267 | Lipid metabolism |
|  |  | Butanoate metabolism | 0.050 | 0.302 | Lipid metabolism |
|  |  | Histidine metabolism | 0.057 | 0.317 |  |
|  |  | Valine, leucine and isoleucine degradation | 0.072 | 0.378 |  |
|  |  | Pantothenate and CoA biosynthesis | 0.077 | 0.381 |  |
|  |  | Primary bile acid biosynthesis | 0.100 | 0.467 |  |
|  |  | Porphyrin and chlorophyll metabolism | 0.166 | 0.734 |  |
|  |  | Ubiquinone and other terpenoid-quinone biosynthesis | 0.201 | 0.803 |  |
|  |  | Vitamin B6 metabolism | 0.201 | 0.803 |  |
|  |  | Purine metabolism | 0.218 | 0.803 |  |
|  |  | Glycerophospholipid metabolism | 0.220 | 0.803 |  |
|  |  | Pyrimidine metabolism | 0.248 | 0.867 |  |
|  |  | Tyrosine metabolism | 0.275 | 0.925 |  |
|  |  | Fructose and mannose metabolism | 0.362 | 1.000 |  |
|  |  | beta-Alanine metabolism | 0.408 | 1.000 |  |
|  |  | Sphingolipid metabolism | 0.408 | 1.000 |  |
|  |  | Galactose metabolism | 0.491 | 1.000 |  |
|  |  | Cysteine and methionine metabolism | 0.563 | 1.000 |  |
|  | WAT | Aminoacyl-tRNA biosynthesis | 0.000 | 0.007 | tRNA metabolism |
|  |  | Alanine, aspartate and glutamate metabolism | 0.003 | 0.080 | Amino acid metabolism |
|  |  | Valine, leucine and isoleucine biosynthesis | 0.003 | 0.080 | Amino acid metabolism |
|  |  | Arginine and proline metabolism | 0.059 | 0.777 |  |
|  |  | Nitrogen metabolism | 0.062 | 0.777 |  |
|  |  | D-Glutamine and D-glutamate metabolism | 0.062 | 0.777 |  |
|  |  | Valine, leucine and isoleucine degradation | 0.065 | 0.777 |  |
|  |  | Taurine and hypotaurine metabolism | 0.082 | 0.777 |  |
|  |  | Primary bile acid biosynthesis | 0.083 | 0.777 |  |
|  |  | Phenylalanine metabolism | 0.120 | 0.945 |  |
|  |  | Arginine biosynthesis | 0.139 | 0.945 |  |
|  |  | Butanoate metabolism | 0.148 | 0.945 |  |
|  |  | Nicotinate and nicotinamide metabolism | 0.148 | 0.945 |  |
|  |  | Histidine metabolism | 0.158 | 0.945 |  |
|  |  | Pantothenate and CoA biosynthesis | 0.184 | 1.000 |  |
|  |  | Selenocompound metabolism | 0.193 | 1.000 |  |
|  |  | Glutathione metabolism | 0.260 | 1.000 |  |
|  |  | Porphyrin and chlorophyll metabolism | 0.276 | 1.000 |  |
|  |  | Glyoxylate and dicarboxylate metabolism | 0.292 | 1.000 |  |
|  |  | Tyrosine metabolism | 0.365 | 1.000 |  |
|  |  | Purine metabolism | 0.513 | 1.000 |  |
| Aerobic capacity*Age | Serum | Valine, leucine and isoleucine biosynthesis | 0.001 | 0.041 | Amino acid metabolism |
|  |  | Aminoacyl-tRNA biosynthesis | 0.001 | 0.041 | tRNA metabolism |
|  |  | Pantothenate and CoA biosynthesis | 0.003 | 0.085 | Metabolism of cofactors and vitamins |
|  |  | Valine, leucine and isoleucine degradation | 0.013 | 0.278 | Amino acid metabolism |
|  |  | Arginine biosynthesis | 0.063 | 0.858 |  |
|  |  | Butanoate metabolism | 0.068 | 0.858 |  |
|  |  | Citrate cycle (TCA cycle) | 0.089 | 0.858 |  |
|  |  | beta-Alanine metabolism | 0.094 | 0.858 |  |
|  |  | Sphingolipid metabolism | 0.094 | 0.858 |  |
|  |  | Propanoate metabolism | 0.102 | 0.858 |  |
|  |  | Alanine, aspartate and glutamate metabolism | 0.123 | 0.940 |  |
|  |  | Glycerophospholipid metabolism | 0.156 | 1.000 |  |
|  |  | Arginine and proline metabolism | 0.164 | 1.000 |  |
|  |  | Pyrimidine metabolism | 0.168 | 1.000 |  |
|  |  | Purine metabolism | 0.269 | 1.000 |  |
|  | Muscle | Arginine and proline metabolism | 0.004 | 0.241 | Amino acid metabolism |
|  |  | Butanoate metabolism | 0.008 | 0.241 | Lipid metabolism |
|  |  | Aminoacyl-tRNA biosynthesis | 0.009 | 0.241 | tRNA metabolism |
|  |  | Alanine, aspartate and glutamate metabolism | 0.026 | 0.442 | Amino acid metabolism |
|  |  | Glutathione metabolism | 0.026 | 0.442 | Amino acid metabolism |
|  |  | Glycine, serine and threonine metabolism | 0.038 | 0.530 | Amino acid metabolism |
|  |  | Synthesis and degradation of ketone bodies | 0.046 | 0.547 | Lipid metabolism |
|  |  | Taurine and hypotaurine metabolism | 0.072 | 0.753 |  |
|  |  | One carbon pool by folate | 0.081 | 0.753 |  |
|  |  | Phenylalanine metabolism | 0.106 | 0.892 |  |
|  |  | Arginine biosynthesis | 0.123 | 0.938 |  |
|  |  | Starch and sucrose metabolism | 0.155 | 0.939 |  |
|  |  | Selenocompound metabolism | 0.171 | 0.939 |  |
|  |  | Citrate cycle (TCA cycle) | 0.171 | 0.939 |  |
|  |  | beta-Alanine metabolism | 0.179 | 0.939 |  |
|  |  | Sphingolipid metabolism | 0.179 | 0.939 |  |
|  |  | Propanoate metabolism | 0.194 | 0.960 |  |
|  |  | Folate biosynthesis | 0.224 | 0.991 |  |
|  |  | Galactose metabolism | 0.224 | 0.991 |  |
|  |  | Glyoxylate and dicarboxylate metabolism | 0.260 | 1.000 |  |
|  |  | Cysteine and methionine metabolism | 0.267 | 1.000 |  |
|  |  | Valine, leucine and isoleucine degradation | 0.315 | 1.000 |  |
|  |  | Tryptophan metabolism | 0.321 | 1.000 |  |
|  |  | Tyrosine metabolism | 0.328 | 1.000 |  |
|  |  | Primary bile acid biosynthesis | 0.353 | 1.000 |  |
|  | WAT | Butanoate metabolism | 0.002 | 0.158 | Lipid metabolism |
|  |  | Glycerophospholipid metabolism | 0.011 | 0.453 | Lipid metabolism |
|  |  | Synthesis and degradation of ketone bodies | 0.023 | 0.644 | Lipid metabolism |
|  |  | Arginine biosynthesis | 0.063 | 1.000 |  |
|  |  | Sphingolipid metabolism | 0.094 | 1.000 |  |
|  |  | Alanine, aspartate and glutamate metabolism | 0.123 | 1.000 |  |
|  |  | Glycine, serine and threonine metabolism | 0.148 | 1.000 |  |
|  |  | Arginine and proline metabolism | 0.164 | 1.000 |  |
|  |  | Pyrimidine metabolism | 0.168 | 1.000 |  |
|  |  | Valine, leucine and isoleucine degradation | 0.172 | 1.000 |  |
|  |  | Tyrosine metabolism | 0.180 | 1.000 |  |
|  |  | Purine metabolism | 0.269 | 1.000 |  |
