# Supplementary material for: Interactive effects of aging and aerobic capacity on energy metabolism–related metabolites of serum, skeletal muscle, and white adipose tissue
Source: GeroScience. 2021 Jun 5;43(6):2679–91. doi: 10.1007/s11357-021-00387-1 (PMC8602622; doi:10.1007/s11357-021-00387-1)
Supplement: Supplementary file 6 — (DOCX 60 kb) [file 11357_2021_387_MOESM5_ESM.docx]

**Supplementary Table 4.** Statistically significant associations between running speed and metabolites in groups of aerobic capacity and age.

|  |  | HCR-Y | | | LCR-Y | | | HCR-O | | | LCR-O | | |
| --- | --- | --- | --- | --- | --- | --- | --- | --- | --- | --- | --- | --- | --- |
| Tissue | Metabolite | Estimate | LowerCI | UpperCI | Estimate | LowerCI | UpperCI | Estimate | LowerCI | UpperCI | Estimate | LowerCI | UpperCI |
|  | Lysine | -0.307 | -3.082 | 2.469 | -0.109 | -3.964 | 3.745 | 1.168 | -3.200 | 5.536 | -0.083 | -2.664 | 2.497 |
|  | Alanine | 5.746 | 0.523 | 10.969 | 1.254 | -1.276 | 3.783 | -8.865 | -14.707 | -3.023 | 0.354 | -2.746 | 3.454 |
|  | Glutamine | -5.141 | -12.829 | 2.547 | -2.794 | -10.198 | 4.611 | 0.496 | -6.468 | 7.461 | 0.039 | -4.938 | 5.015 |
|  | Betaine | 1.293 | -3.481 | 6.067 | 1.551 | -3.357 | 6.459 | 0.807 | -3.126 | 4.740 | -0.096 | -2.025 | 1.834 |
|  | Creatine | 6.787 | -1.536 | 15.110 | 4.320 | -3.281 | 11.921 | -0.531 | -4.646 | 3.583 | -0.117 | -2.554 | 2.321 |
|  | Taurine | -4.203 | -8.762 | 0.355 | -2.315 | -7.750 | 3.120 | -3.642 | -6.432 | -0.853 | -0.209 | -2.454 | 2.036 |
|  | Glyceraldehyde | 5.875 | -3.022 | 14.773 | 3.444 | -2.741 | 9.629 | 0.785 | -2.768 | 4.338 | 0.159 | -2.671 | 2.989 |
|  | Serine | -1.585 | -3.562 | 0.393 | -0.342 | -4.154 | 3.469 | 1.063 | -3.951 | 6.076 | 0.201 | -2.512 | 2.915 |
|  | Threonine | 0.148 | -2.963 | 3.259 | -0.962 | -3.440 | 1.517 | 2.016 | -3.121 | 7.152 | 0.053 | -2.498 | 2.605 |
|  | Allantoin | 2.292 | -0.437 | 5.022 | 0.331 | -2.856 | 3.517 | -0.381 | -3.390 | 2.627 | 0.646 | -2.079 | 3.371 |
|  | Hippuric.acid | 4.439 | -1.432 | 10.311 | -0.841 | -6.539 | 4.856 | 7.306 | -4.004 | 18.616 | 0.464 | -10.828 | 11.755 |
|  | Leucine | 3.459 | -6.141 | 13.058 | 2.121 | -3.623 | 7.865 | 0.899 | -32.228 | 34.025 | 0.639 | -9.479 | 10.756 |
|  | Chenodeoxycholic acid | -2.194 | -5.955 | 1.567 | -1.310 | -4.232 | 1.611 | -0.069 | -2.620 | 2.482 | -0.122 | -2.851 | 2.606 |
|  | Valine | -0.994 | -6.449 | 4.460 | 1.822 | -2.167 | 5.811 | 3.607 | -1.174 | 8.387 | 1.002 | -3.419 | 5.423 |
|  | L-glutamic acid | 1.009 | -1.695 | 3.713 | 1.255 | -2.066 | 4.576 | -10.747 | -21.766 | 0.272 | 1.238 | -6.156 | 8.632 |
|  | Glycine | -0.659 | -3.030 | 1.712 | -1.303 | -8.371 | 5.765 | -0.374 | -4.023 | 3.274 | 0.678 | -4.526 | 5.882 |
|  | Citrulline | 0.772 | -2.230 | 3.774 | -0.467 | -5.025 | 4.092 | 4.678 | -2.477 | 11.832 | 0.279 | -6.857 | 7.415 |
|  | Proline | -2.189 | -7.298 | 2.919 | -2.492 | -7.930 | 2.945 | 1.959 | -1.967 | 5.884 | -0.020 | -2.383 | 2.343 |
|  | Choline | -2.607 | -7.221 | 2.008 | 2.518 | -2.954 | 7.989 | -2.786 | -6.393 | 0.821 | -0.434 | -2.973 | 2.106 |
|  | Ornithine | 5.759 | -2.217 | 13.735 | -2.078 | -7.788 | 3.631 | -1.015 | -5.784 | 3.753 | 0.155 | -3.254 | 3.563 |
|  | Hydroxyproline | 5.146 | -7.575 | 17.867 | 5.063 | -4.934 | 15.061 | -8.014 | -33.264 | 17.236 | 0.503 | -9.551 | 10.557 |
|  | Histidine | -0.256 | -3.792 | 3.280 | -0.086 | -2.687 | 2.516 | -2.841 | -8.476 | 2.794 | 0.003 | -5.669 | 5.674 |
|  | Isoleucine | -3.298 | -10.447 | 3.852 | 0.673 | -2.713 | 4.059 | -3.693 | -7.226 | -0.161 | 0.070 | -2.030 | 2.169 |
|  | Tryptophan | 4.676 | 0.598 | 8.753 | 0.380 | -2.287 | 3.046 | 4.151 | 1.342 | 6.959 | -0.105 | -2.061 | 1.851 |
|  | Myoinositol | -0.500 | -5.753 | 4.753 | -0.900 | -4.239 | 2.438 | -7.159 | -13.259 | -1.059 | -0.045 | -5.127 | 5.036 |
|  | Tyrosine | -2.198 | -5.280 | 0.884 | 0.674 | -2.549 | 3.896 | -2.641 | -4.848 | -0.434 | 0.120 | -2.149 | 2.389 |
|  | Phenylalanine | -3.404 | -11.996 | 5.187 | 2.461 | -3.051 | 7.973 | -2.940 | -5.989 | 0.109 | 0.051 | -1.943 | 2.045 |
|  | Asparagine | -0.795 | -2.779 | 1.189 | -1.529 | -4.189 | 1.130 | 2.791 | -4.309 | 9.891 | 0.503 | -2.990 | 3.996 |
|  | Carnitine | 2.547 | -0.234 | 5.328 | 1.622 | -1.102 | 4.347 | 0.951 | -7.544 | 9.445 | 0.334 | -2.244 | 2.912 |
|  | Acetoacetic acid | -1.202 | -4.973 | 2.568 | -1.966 | -7.283 | 3.351 | 19.827 | -7.248 | 46.902 | -2.345 | -16.667 | 11.977 |
|  | Creatinine | 1.930 | -1.419 | 5.278 | 1.042 | -2.212 | 4.296 | 6.520 | -3.532 | 16.572 | 1.062 | -4.820 | 6.944 |
|  | Succinate | 3.862 | -2.645 | 10.369 | 1.582 | -1.599 | 4.763 | -4.032 | -6.390 | -1.674 | 0.060 | -2.424 | 2.543 |
|  | L-methionine | -1.552 | -3.471 | 0.368 | -0.318 | -3.374 | 2.738 | 2.454 | -4.288 | 9.197 | 0.188 | -3.219 | 3.594 |
|  | deoxycytidine | 3.201 | -1.285 | 7.687 | 1.777 | -3.640 | 7.194 | -0.245 | -3.725 | 3.236 | -0.119 | -1.924 | 1.686 |
|  | Arginine | 1.165 | -2.377 | 4.708 | 0.727 | -2.303 | 3.757 | -0.462 | -3.004 | 2.080 | 0.193 | -4.189 | 4.575 |
|  | Aspartate | 1.258 | -1.285 | 3.802 | 1.774 | -9.938 | 13.485 | -1.836 | -6.114 | 2.443 | 0.444 | -3.061 | 3.949 |
|  | Homocysteine | 2.081 | -1.437 | 5.599 | -1.166 | -5.761 | 3.429 | -0.706 | -3.104 | 1.692 | -0.398 | -3.287 | 2.492 |
| Serum | Acetylcarnitine | -1.755 | -5.054 | 1.543 | 0.578 | -1.650 | 2.807 | -1.727 | -5.643 | 2.188 | 0.569 | -2.406 | 3.545 |
|  | Uracil | -3.548 | -6.299 | -0.797 | 1.544 | -2.576 | 5.664 | -8.875 | -12.163 | -5.588 | -0.355 | -3.976 | 3.266 |
|  | Niacinamide | -2.908 | -5.683 | -0.133 | 0.899 | -1.385 | 3.182 | 11.434 | -7.859 | 30.728 | -1.418 | -8.712 | 5.877 |
|  | D-glucuronic acid | 2.898 | -1.058 | 6.853 | -0.947 | -4.260 | 2.367 | -1.573 | -4.331 | 1.185 | 0.352 | -2.467 | 3.171 |
|  | D-ribose-5-phosphate | -0.312 | -2.935 | 2.310 | -1.625 | -5.025 | 1.775 | -6.651 | -16.316 | 3.014 | -0.272 | -7.208 | 6.664 |
|  | Sorbitol | -0.291 | -2.889 | 2.307 | -0.939 | -4.266 | 2.388 | -2.424 | -6.990 | 2.142 | -0.029 | -2.946 | 2.888 |
|  | Phosphoethanolamine | 2.337 | -4.037 | 8.711 | 4.097 | -2.339 | 10.532 | 1.511 | -1.647 | 4.670 | -0.332 | -4.128 | 3.464 |
|  | DimethylGlycine | 19.935 | -9.838 | 49.707 | -9.239 | -33.430 | 14.953 | -1.702 | -5.125 | 1.721 | -0.555 | -3.767 | 2.656 |
|  | 2-aminoisobutyric acid | 1.104 | -1.603 | 3.812 | 0.018 | -2.677 | 2.714 | 6.605 | 1.916 | 11.293 | -0.085 | -2.992 | 2.822 |
|  | Spermidine | 1.520 | -2.500 | 5.540 | -1.556 | -5.035 | 1.922 | 1.414 | -2.244 | 5.071 | -0.175 | -2.546 | 2.196 |
|  | Pantothenic acid | 0.002 | -2.768 | 2.773 | -0.612 | -3.612 | 2.387 | -0.140 | -5.364 | 5.083 | 0.322 | -3.113 | 3.756 |
|  | Aminodipic acid | 5.199 | 0.766 | 9.632 | -0.158 | -5.550 | 5.234 | -0.875 | -8.460 | 6.711 | 0.922 | -6.138 | 7.981 |
|  | Carnosine | 16.337 | 1.199 | 31.475 | 1.024 | -4.434 | 6.482 | -16.003 | -27.778 | -4.229 | 0.545 | -7.824 | 8.915 |
|  | Asymmetric dimethylarginine | 0.653 | -1.625 | 2.930 | 2.006 | -1.428 | 5.441 | -5.343 | -9.256 | -1.430 | -0.092 | -2.805 | 2.620 |
|  | Guanidinoacetic acid | -0.387 | -3.369 | 2.595 | 0.413 | -5.120 | 5.946 | -2.501 | -6.723 | 1.720 | 0.392 | -1.743 | 2.528 |
|  | Taurocholic acid | -3.102 | -7.173 | 0.970 | -1.776 | -7.213 | 3.660 | -0.589 | -5.945 | 4.767 | 1.070 | -6.528 | 8.668 |
|  | L-kynurenine | 0.385 | -3.425 | 4.194 | 2.118 | -1.822 | 6.058 | 6.177 | 1.409 | 10.945 | 0.106 | -2.313 | 2.525 |
|  | Trimethylamine-N-oxide | 0.994 | -2.033 | 4.021 | 0.272 | -3.494 | 4.038 | 5.394 | 1.592 | 9.196 | 0.641 | -1.258 | 2.541 |
|  | Symmetric dimethylarginine | -0.606 | -3.545 | 2.332 | 0.767 | -1.827 | 3.361 | -3.075 | -6.301 | 0.150 | 0.209 | -2.273 | 2.691 |
|  | 2-deoxyuridine | 2.174 | -0.850 | 5.197 | 2.883 | -1.373 | 7.139 | -0.301 | -4.234 | 3.631 | -0.014 | -2.365 | 2.338 |
|  | Sucrose | 1.030 | -2.574 | 4.633 | -0.835 | -4.238 | 2.567 | -1.105 | -3.892 | 1.682 | 0.180 | -2.151 | 2.512 |
|  | Xanthosine | 0.409 | -2.651 | 3.468 | 1.644 | -1.738 | 5.025 | -3.081 | -6.372 | 0.210 | 0.513 | -1.686 | 2.713 |
|  | IMP | 5.050 | -0.581 | 10.681 | -1.368 | -5.324 | 2.588 | -1.040 | -4.656 | 2.577 | -0.291 | -3.511 | 2.930 |
|  | Propionylcarnitine | -2.126 | -4.653 | 0.401 | 0.970 | -1.492 | 3.432 | 2.420 | -1.525 | 6.365 | 0.058 | -3.350 | 3.466 |
|  | 5-hydroxyindole-3-acetic acid | -6.873 | -14.788 | 1.042 | 0.892 | -4.283 | 6.067 | 5.890 | 1.754 | 10.027 | 0.565 | -2.158 | 3.287 |
|  | Glycocholic acid | -3.086 | -8.764 | 2.591 | 1.587 | -8.684 | 11.859 | 0.790 | -2.123 | 3.703 | -0.433 | -2.346 | 1.479 |
|  | Isobutyrylcarnitine | 0.283 | -4.193 | 4.759 | 1.337 | -1.935 | 4.608 | 2.051 | -9.568 | 13.669 | 0.884 | -6.083 | 7.852 |
|  | Taurochenodeoxycholic acid | -1.808 | -4.183 | 0.567 | -1.304 | -4.560 | 1.951 | 0.988 | -2.753 | 4.728 | -0.412 | -4.423 | 3.599 |
|  | Cystathionine | -0.260 | -2.690 | 2.171 | 0.319 | -3.267 | 3.905 | -4.153 | -7.725 | -0.580 | 0.535 | -1.621 | 2.690 |
|  | Cytidine | 1.060 | -2.448 | 4.569 | 0.770 | -1.835 | 3.374 | -1.218 | -6.461 | 4.025 | 0.663 | -2.716 | 4.041 |
|  | Cholic acid | 0.787 | -2.542 | 4.115 | -0.839 | -3.119 | 1.442 | -0.227 | -4.348 | 3.893 | -0.899 | -3.684 | 1.886 |
|  | GABA | -0.186 | -3.132 | 2.759 | -0.444 | -3.759 | 2.871 | -3.410 | -9.267 | 2.448 | 0.272 | -3.290 | 3.834 |
|  | Gamma-glutamylcysteine | -6.542 | -12.860 | -0.223 | 1.498 | -6.173 | 9.169 | 1.648 | -0.373 | 3.670 | 0.086 | -1.742 | 1.913 |
|  | Octanoylcarnitine | 1.157 | -1.246 | 3.561 | 0.890 | -1.272 | 3.051 | -0.500 | -5.043 | 4.044 | -0.822 | -5.170 | 3.526 |
|  | Kynurenic acid | 1.003 | -1.736 | 3.741 | -0.921 | -5.716 | 3.873 | 7.723 | -0.148 | 15.595 | -1.585 | -14.061 | 10.891 |
|  | Isovalerylcarnitine | -0.685 | -6.152 | 4.783 | 1.918 | -0.703 | 4.539 | 4.473 | -6.879 | 15.825 | 0.381 | -2.753 | 3.514 |
|  | AMP | 1.989 | -1.716 | 5.693 | 0.078 | -1.961 | 2.117 | -1.082 | -5.164 | 3.000 | -0.131 | -3.512 | 3.250 |
|  | Decanoylcarnitine | 0.012 | -2.775 | 2.798 | 1.095 | -1.350 | 3.539 | 4.525 | -0.747 | 9.797 | -0.262 | -2.477 | 1.953 |
|  | 4-Pyridoxic acid | 1.976 | -0.517 | 4.468 | 1.323 | -2.305 | 4.950 | 10.847 | 0.950 | 20.744 | 1.168 | -7.897 | 10.234 |
|  | Folic acid | 3.527 | -1.730 | 8.785 | 1.318 | -4.128 | 6.764 | -47.317 | -93.532 | -1.102 | -1.315 | -28.661 | 26.030 |
|  | Inosine | -3.176 | -8.331 | 1.979 | 1.437 | -1.875 | 4.749 | -2.076 | -4.337 | 0.185 | -0.052 | -2.547 | 2.443 |
|  | NAD | 0.034 | -4.247 | 4.315 | -1.265 | -6.950 | 4.420 | -2.753 | -8.698 | 3.191 | -0.105 | -3.425 | 3.215 |
|  | Nicotinic.Acid | 0.213 | -3.778 | 4.203 | 3.181 | -8.682 | 15.043 | 0.916 | -1.838 | 3.670 | 0.004 | -2.166 | 2.175 |
|  | Adenosine | -2.238 | -5.177 | 0.701 | -1.337 | -5.360 | 2.687 | -2.273 | -6.446 | 1.901 | -0.250 | -2.916 | 2.416 |
|  | Hexanoylcarnitine | 0.385 | -4.017 | 4.787 | 0.937 | -1.416 | 3.290 | 0.978 | -3.027 | 4.984 | 0.311 | -3.081 | 3.703 |
|  | Xanthine | -0.089 | -11.876 | 11.698 | 3.014 | -5.943 | 11.970 | -2.455 | -12.055 | 7.144 | -1.727 | -9.632 | 6.178 |
|  | 1-methylhistamine | 1.554 | -1.167 | 4.275 | -0.341 | -3.132 | 2.450 | -11.024 | -17.602 | -4.445 | -0.192 | -2.258 | 1.874 |
|  | Cytosine | 1.709 | -3.803 | 7.222 | -0.315 | -3.012 | 2.382 | -0.490 | -4.422 | 3.441 | -0.254 | -2.693 | 2.185 |
|  | Adenine | -0.997 | -4.212 | 2.218 | 2.081 | -2.164 | 6.326 | -0.972 | -10.120 | 8.175 | -0.460 | -8.345 | 7.425 |
|  | Alanine | 3.901 | -23.176 | 30.977 | 11.085 | -15.157 | 37.327 | -160.183 | -332.688 | 12.321 | 2.998 | -50.147 | 56.144 |
|  | Taurine | -1.479 | -10.384 | 7.427 | -2.100 | -15.942 | 11.742 | 30.868 | -8.714 | 70.450 | -3.564 | -18.782 | 11.653 |
|  | Hydroxyproline | 0.753 | -18.841 | 20.347 | 6.690 | -15.203 | 28.583 | -92.478 | -450.074 | 265.118 | 14.467 | -154.432 | 183.367 |
|  | Creatine | 1.597 | -2.179 | 5.372 | -0.665 | -4.342 | 3.012 | 0.074 | -3.692 | 3.839 | 0.697 | -1.718 | 3.113 |
|  | Glutamine | 6.567 | -10.584 | 23.719 | -2.224 | -14.807 | 10.359 | 0.596 | -6.053 | 7.245 | -2.110 | -11.704 | 7.484 |
|  | Acetoacetic acid | -0.352 | -5.224 | 4.521 | 2.962 | -4.277 | 10.202 | -40.712 | -67.742 | -13.683 | 5.948 | -8.621 | 20.517 |
|  | Glycine | 0.305 | -3.738 | 4.348 | -7.848 | -19.157 | 3.460 | -6.988 | -13.592 | -0.383 | 0.702 | -2.876 | 4.279 |
|  | Niacinamide | 0.246 | -3.717 | 4.210 | -1.438 | -5.085 | 2.210 | -8.233 | -12.814 | -3.653 | 0.892 | -2.514 | 4.299 |
|  | Carnitine | -0.795 | -4.624 | 3.034 | 1.049 | -4.133 | 6.232 | 4.604 | -4.870 | 14.077 | 0.299 | -1.237 | 1.835 |
|  | Serine | -4.384 | -13.007 | 4.240 | -1.684 | -7.013 | 3.646 | 4.952 | -3.632 | 13.536 | -0.496 | -6.139 | 5.148 |
|  | Threonine | -0.069 | -4.515 | 4.377 | -1.754 | -5.314 | 1.806 | 4.032 | -3.599 | 11.662 | 0.113 | -5.439 | 5.666 |
|  | Acetylcarnitine | -1.051 | -4.588 | 2.486 | -1.201 | -3.344 | 0.942 | -9.142 | -13.339 | -4.944 | -0.716 | -3.134 | 1.701 |
|  | L-glutamic acid | 3.403 | -2.412 | 9.219 | 0.553 | -5.565 | 6.670 | 3.985 | -3.620 | 11.590 | 1.409 | -5.985 | 8.804 |
|  | Choline | 0.552 | -2.715 | 3.820 | -2.348 | -9.329 | 4.633 | -5.623 | -10.702 | -0.543 | -0.640 | -2.990 | 1.710 |
|  | Glutathione | 1.775 | -2.895 | 6.446 | -0.224 | -10.163 | 9.716 | -21.525 | -34.487 | -8.562 | 1.134 | -4.253 | 6.521 |
|  | Creatinine | -8.672 | -46.256 | 28.912 | 14.606 | -31.006 | 60.217 | 4.636 | -0.650 | 9.923 | -0.763 | -4.212 | 2.686 |
|  | Proline | -3.529 | -9.881 | 2.824 | -3.995 | -10.812 | 2.821 | 2.439 | -2.488 | 7.365 | 0.239 | -3.652 | 4.130 |
|  | Hypoxanthine | -4.276 | -20.170 | 11.618 | 3.662 | -9.186 | 16.509 | -4.197 | -6.993 | -1.401 | 0.499 | -1.653 | 2.651 |
|  | Valine | -1.806 | -7.644 | 4.032 | -1.772 | -7.263 | 3.718 | -3.400 | -9.658 | 2.859 | 0.891 | -2.342 | 4.123 |
|  | Succinate | -1.863 | -8.364 | 4.638 | 1.388 | -9.758 | 12.534 | -1.360 | -9.917 | 7.197 | 0.369 | -3.601 | 4.339 |
|  | Leucine | -2.812 | -7.294 | 1.670 | -1.055 | -5.862 | 3.752 | -8.173 | -13.585 | -2.762 | 0.254 | -1.610 | 2.117 |
|  | Inosine | 0.050 | -3.849 | 3.948 | 1.085 | -1.544 | 3.713 | -3.760 | -6.932 | -0.588 | 0.753 | -1.905 | 3.411 |
|  | Glyceraldehyde | -0.869 | -3.751 | 2.014 | -1.230 | -5.943 | 3.483 | -2.697 | -6.586 | 1.192 | 0.298 | -2.274 | 2.869 |
|  | Allantoin | -1.774 | -7.474 | 3.925 | 0.391 | -4.498 | 5.279 | -4.049 | -13.692 | 5.594 | 0.229 | -3.090 | 3.548 |
|  | Betaine | 0.850 | -4.716 | 6.416 | -0.853 | -3.891 | 2.185 | -1.517 | -5.293 | 2.259 | -0.752 | -5.035 | 3.531 |
|  | Citrulline | 3.970 | -2.006 | 9.946 | 0.880 | -3.667 | 5.428 | -1.206 | -6.822 | 4.409 | -0.761 | -8.014 | 6.492 |
|  | Asparagine | -2.030 | -12.835 | 8.776 | -0.066 | -9.334 | 9.202 | 11.629 | -24.879 | 48.137 | 3.193 | -6.221 | 12.608 |
|  | Tyrosine | 1.194 | -3.067 | 5.454 | 1.980 | -4.627 | 8.586 | -3.669 | -7.355 | 0.017 | 0.480 | -2.381 | 3.342 |
|  | Aspartate | -0.834 | -4.154 | 2.486 | 0.745 | -2.264 | 3.754 | 12.348 | 3.308 | 21.388 | 1.459 | -2.821 | 5.740 |
|  | Isoleucine | -3.356 | -12.467 | 5.756 | -2.537 | -9.544 | 4.470 | -6.043 | -11.634 | -0.453 | 0.237 | -2.651 | 3.125 |
|  | Ornithine | -0.666 | -4.809 | 3.476 | 1.789 | -3.355 | 6.932 | 1.605 | -6.406 | 9.617 | 1.447 | -3.739 | 6.634 |
|  | Phenylalanine | -0.095 | -8.250 | 8.059 | -3.514 | -14.245 | 7.218 | -4.497 | -9.703 | 0.708 | 0.399 | -2.557 | 3.354 |
|  | L-methionine | 0.581 | -3.856 | 5.018 | -2.273 | -6.991 | 2.445 | -13.777 | -21.421 | -6.133 | 0.941 | -2.014 | 3.896 |
|  | Uracil | -0.316 | -3.991 | 3.360 | 0.441 | -2.318 | 3.201 | -2.267 | -4.723 | 0.188 | -0.597 | -3.111 | 1.917 |
| Muscle | Tryptophan | 2.464 | -3.348 | 8.276 | -0.698 | -4.777 | 3.381 | -4.284 | -9.638 | 1.070 | 0.851 | -3.573 | 5.275 |
|  | Xanthine | -1.223 | -6.164 | 3.717 | -1.012 | -5.196 | 3.171 | -2.507 | -4.704 | -0.311 | 0.544 | -1.434 | 2.521 |
|  | IMP | 1.695 | -2.154 | 5.544 | 1.387 | -2.038 | 4.813 | -2.850 | -4.706 | -0.995 | 0.234 | -1.755 | 2.223 |
|  | Pantothenic acid | 3.405 | -4.974 | 11.785 | -0.316 | -5.825 | 5.194 | -0.739 | -11.254 | 9.776 | 1.223 | -3.387 | 5.833 |
|  | Chenodeoxycholic acid | -2.537 | -10.202 | 5.128 | 2.480 | -14.818 | 19.777 | -0.323 | -3.054 | 2.408 | 0.287 | -1.892 | 2.465 |
|  | Sorbitol | -2.107 | -18.969 | 14.755 | 9.667 | -5.062 | 24.397 | -5.877 | -8.988 | -2.766 | 0.958 | -2.059 | 3.975 |
|  | Hippuric.acid | 6.188 | -0.427 | 12.802 | -0.583 | -7.984 | 6.819 | 6.283 | -3.255 | 15.822 | -0.209 | -3.419 | 3.001 |
|  | deoxycytidine | 2.454 | -2.840 | 7.748 | 3.341 | -1.494 | 8.175 | 3.598 | -0.283 | 7.478 | -0.195 | -1.920 | 1.530 |
|  | Taurocholic acid | -4.130 | -7.974 | -0.285 | -0.585 | -2.875 | 1.705 | -3.521 | -6.396 | -0.646 | 0.387 | -1.618 | 2.392 |
|  | 2-aminoisobutyric acid | 0.543 | -1.444 | 2.531 | -0.084 | -2.415 | 2.248 | 5.360 | 1.614 | 9.105 | -0.621 | -3.809 | 2.567 |
|  | Cytidine | -0.536 | -9.238 | 8.166 | -0.961 | -4.067 | 2.145 | -5.910 | -9.783 | -2.037 | 0.150 | -1.278 | 1.577 |
|  | Octanoylcarnitine | 2.349 | -1.565 | 6.264 | 2.118 | -3.030 | 7.265 | -0.617 | -4.164 | 2.930 | -0.485 | -3.158 | 2.187 |
|  | Phosphoethanolamine | -0.204 | -3.127 | 2.718 | -0.442 | -3.032 | 2.149 | -2.966 | -7.337 | 1.404 | -0.038 | -2.914 | 2.838 |
|  | Isobutyrylcarnitine | 1.859 | -2.029 | 5.747 | 1.741 | -3.075 | 6.558 | -2.365 | -5.146 | 0.416 | -0.850 | -3.848 | 2.148 |
|  | Folic acid | 0.486 | -8.994 | 9.966 | 6.324 | -6.175 | 18.822 | 0.957 | -14.458 | 16.371 | 2.037 | -7.582 | 11.656 |
|  | Hexanoylcarnitine | 0.773 | -1.461 | 3.008 | 1.368 | -2.128 | 4.865 | -0.604 | -3.673 | 2.464 | -0.525 | -3.500 | 2.451 |
|  | Decanoylcarnitine | 2.387 | -3.577 | 8.350 | 1.770 | -3.798 | 7.338 | -0.823 | -5.813 | 4.167 | 0.027 | -4.033 | 4.087 |
|  | Guanidinoacetic acid | 1.402 | -8.066 | 10.870 | -1.428 | -12.771 | 9.915 | -5.400 | -11.025 | 0.225 | -0.259 | -3.210 | 2.692 |
|  | DimethylGlycine | 0.173 | -2.899 | 3.245 | 0.847 | -2.277 | 3.971 | -2.892 | -9.469 | 3.685 | -1.797 | -9.082 | 5.487 |
|  | Sucrose | 5.212 | -0.358 | 10.781 | -1.064 | -4.864 | 2.737 | -1.708 | -7.745 | 4.329 | 1.243 | -4.031 | 6.517 |
|  | L-kynurenine | -0.570 | -2.694 | 1.555 | 1.124 | -1.497 | 3.745 | -1.160 | -6.957 | 4.638 | 0.339 | -2.396 | 3.075 |
|  | Trimethylamine-N-oxide | -3.546 | -8.816 | 1.724 | -1.112 | -5.285 | 3.061 | 2.917 | 0.426 | 5.408 | 0.394 | -1.684 | 2.472 |
|  | Propionylcarnitine | 2.659 | -1.829 | 7.148 | 2.001 | -1.587 | 5.588 | -5.169 | -10.160 | -0.177 | -0.224 | -2.811 | 2.362 |
|  | NAD | -2.115 | -7.916 | 3.686 | 0.681 | -5.136 | 6.499 | 0.027 | -6.454 | 6.509 | 0.243 | -2.150 | 2.636 |
|  | Isovalerylcarnitine | 5.648 | -0.099 | 11.395 | 0.886 | -1.445 | 3.217 | -12.717 | -21.246 | -4.188 | -0.223 | -2.282 | 1.836 |
|  | GABA | 3.146 | 0.018 | 6.275 | 1.921 | -4.285 | 8.127 | -7.522 | -12.924 | -2.119 | 1.231 | -2.846 | 5.308 |
|  | Spermidine | -0.530 | -6.069 | 5.009 | 1.922 | -5.820 | 9.663 | -4.910 | -17.464 | 7.644 | 2.204 | -4.856 | 9.264 |
|  | Glycocholic acid | 3.262 | -1.226 | 7.750 | 3.432 | -2.434 | 9.299 | -2.792 | -6.286 | 0.701 | -0.180 | -2.119 | 1.759 |
|  | 2-deoxyuridine | -1.367 | -5.606 | 2.872 | 0.889 | -3.281 | 5.060 | 0.422 | -4.339 | 5.182 | 0.119 | -2.467 | 2.706 |
|  | AMP | -5.937 | -13.025 | 1.152 | 1.137 | -2.466 | 4.740 | -6.859 | -10.756 | -2.961 | 0.559 | -4.852 | 5.971 |
|  | Adenosine | -4.786 | -15.437 | 5.864 | 1.308 | -5.553 | 8.169 | 4.055 | -2.528 | 10.637 | -1.579 | -7.194 | 4.036 |
|  | Adenine | -9.464 | -19.613 | 0.686 | 2.147 | -4.618 | 8.912 | -1.484 | -22.455 | 19.488 | 0.264 | -7.449 | 7.977 |
|  | Cytosine | 2.505 | -0.603 | 5.614 | -1.337 | -8.648 | 5.974 | 0.511 | -4.175 | 5.197 | -0.250 | -3.072 | 2.572 |
|  | Kynurenic acid | -0.318 | -3.240 | 2.604 | 1.250 | -2.245 | 4.744 | -0.247 | -4.473 | 3.979 | 0.386 | -1.701 | 2.474 |
|  | Taurochenodeoxycholic acid | -0.949 | -3.525 | 1.627 | 1.137 | -2.910 | 5.184 | 0.500 | -2.792 | 3.792 | 0.377 | -2.361 | 3.116 |
|  | Normetanephrine | 1.349 | -2.672 | 5.370 | -0.651 | -6.007 | 4.706 | -1.925 | -7.637 | 3.788 | -1.514 | -7.800 | 4.771 |
|  | 4-pyridoxic acid | 1.438 | -1.273 | 4.149 | 1.260 | -3.371 | 5.890 | -2.725 | -13.260 | 7.810 | 1.266 | -5.044 | 7.575 |
|  | Taurine | -4.673 | -12.784 | 3.439 | -0.901 | -5.835 | 4.034 | -10.349 | -22.891 | 2.194 | 0.934 | -7.809 | 9.677 |
|  | Alanine | 21.644 | -9.772 | 53.060 | 3.480 | -11.148 | 18.108 | -19.802 | -38.175 | -1.429 | -0.095 | -17.844 | 17.654 |
|  | Choline | -4.656 | -12.985 | 3.673 | -1.487 | -7.251 | 4.278 | 4.633 | -1.458 | 10.723 | -0.811 | -5.668 | 4.047 |
|  | Acetoacetic acid | -3.197 | -13.477 | 7.084 | -2.636 | -12.148 | 6.876 | 2.832 | -9.701 | 15.365 | -3.341 | -27.505 | 20.823 |
|  | Myoinositol | -0.601 | -6.897 | 5.696 | -1.000 | -4.674 | 2.673 | -4.259 | -28.433 | 19.915 | 0.606 | -6.120 | 7.331 |
|  | Glutathione | -6.051 | -13.729 | 1.626 | -1.133 | -6.380 | 4.114 | 6.252 | -0.928 | 13.433 | 0.630 | -3.060 | 4.321 |
|  | Hypoxanthine | -1.569 | -11.959 | 8.820 | -0.608 | -4.951 | 3.734 | 3.635 | -4.062 | 11.333 | -0.687 | -4.796 | 3.422 |
|  | Creatine | 4.469 | -5.397 | 14.335 | 0.819 | -2.803 | 4.441 | -10.164 | -21.034 | 0.706 | -0.330 | -3.866 | 3.206 |
|  | Hydroxyproline | 32.596 | -22.807 | 87.998 | 4.124 | -15.161 | 23.410 | 14.592 | -17.088 | 46.272 | 1.463 | -21.481 | 24.407 |
|  | Glutamine | 1.896 | -6.796 | 10.588 | 0.102 | -5.909 | 6.114 | 3.386 | -2.275 | 9.047 | 0.601 | -3.249 | 4.450 |
|  | Glycine | 3.150 | -2.398 | 8.699 | 0.256 | -3.347 | 3.858 | -5.552 | -15.508 | 4.403 | 0.747 | -5.891 | 7.385 |
|  | L-glutamic acid | 5.623 | -8.015 | 19.261 | -0.566 | -6.898 | 5.765 | 0.019 | -5.585 | 5.622 | -0.777 | -5.387 | 3.833 |
|  | Serine | -0.020 | -3.792 | 3.751 | -2.092 | -9.371 | 5.188 | -5.185 | -15.543 | 5.173 | 0.257 | -4.232 | 4.745 |
|  | Succinate | 2.734 | -3.130 | 8.598 | -0.359 | -3.950 | 3.233 | 4.411 | -10.482 | 19.304 | -0.650 | -6.289 | 4.990 |
|  | Threonine | 1.106 | -3.338 | 5.551 | -1.346 | -5.193 | 2.501 | 8.407 | 2.069 | 14.745 | 2.155 | -7.667 | 11.976 |
|  | Inosine | -0.234 | -9.138 | 8.669 | -1.201 | -6.830 | 4.429 | 2.690 | -3.639 | 9.020 | -0.538 | -4.472 | 3.395 |
|  | Ornithine | 1.009 | -6.718 | 8.737 | -0.715 | -4.901 | 3.471 | -1.332 | -7.474 | 4.811 | 0.856 | -4.326 | 6.039 |
|  | Niacinamide | -2.006 | -7.376 | 3.363 | -1.144 | -5.218 | 2.930 | 6.296 | -3.814 | 16.406 | -0.905 | -10.932 | 9.122 |
|  | Chenodeoxycholic acid | 1.416 | -20.782 | 23.614 | -0.672 | -7.955 | 6.611 | 22.652 | 12.189 | 33.115 | 3.652 | -11.376 | 18.679 |
|  | Proline | 0.931 | -4.513 | 6.376 | -1.383 | -5.681 | 2.915 | -2.537 | -9.184 | 4.111 | 0.253 | -4.836 | 5.342 |
|  | Leucine | 0.507 | -3.788 | 4.802 | -0.912 | -5.272 | 3.448 | -21.094 | -38.300 | -3.889 | -1.183 | -10.893 | 8.528 |
|  | Valine | 0.596 | -8.367 | 9.560 | -2.268 | -11.358 | 6.822 | -4.326 | -12.960 | 4.309 | -0.169 | -4.588 | 4.250 |
|  | Uracil | 3.605 | -5.497 | 12.708 | -0.552 | -4.252 | 3.148 | 0.545 | -7.048 | 8.139 | -0.839 | -6.160 | 4.481 |
|  | Arginine | -4.529 | -21.997 | 12.939 | -0.210 | -11.258 | 10.839 | 0.051 | -3.015 | 3.118 | 1.044 | -4.038 | 6.125 |
|  | Adenosine | -1.826 | -7.841 | 4.188 | -0.193 | -3.484 | 3.098 | 40.576 | 9.443 | 71.708 | 0.627 | -13.033 | 14.286 |
|  | Betaine | 0.446 | -5.087 | 5.979 | -0.916 | -5.606 | 3.775 | 2.787 | -4.631 | 10.205 | 0.122 | -7.170 | 7.414 |
|  | Aspartate | -0.255 | -4.171 | 3.660 | -1.074 | -6.188 | 4.040 | -0.048 | -11.017 | 10.920 | 0.534 | -5.373 | 6.441 |
|  | Tyrosine | 0.557 | -3.916 | 5.029 | -0.500 | -5.024 | 4.023 | -10.953 | -29.627 | 7.722 | -0.196 | -4.870 | 4.478 |
|  | Xanthine | 2.904 | -5.544 | 11.352 | -1.184 | -5.861 | 3.494 | 5.385 | -0.911 | 11.680 | -0.648 | -4.497 | 3.200 |
|  | Asparagine | 1.028 | -8.236 | 10.291 | -2.841 | -14.140 | 8.459 | -3.114 | -11.468 | 5.239 | 1.339 | -5.481 | 8.159 |
|  | Hippuric acid | 6.086 | -1.337 | 13.510 | -1.082 | -4.341 | 2.177 | 6.425 | 1.363 | 11.487 | -0.063 | -6.426 | 6.300 |
|  | Carnitine | 0.866 | -4.229 | 5.962 | 1.172 | -5.311 | 7.656 | -6.346 | -11.550 | -1.142 | 0.973 | -5.078 | 7.024 |
|  | Acetylcarnitine | 0.000 | -4.544 | 4.544 | 1.349 | -4.015 | 6.713 | -3.026 | -6.912 | 0.860 | 0.214 | -6.215 | 6.643 |
|  | Pantothenic acid | -2.472 | -5.130 | 0.185 | -1.542 | -4.784 | 1.700 | 12.729 | 7.569 | 17.889 | 0.757 | -4.265 | 5.779 |
| WAT | Phenylalanine | 1.260 | -5.027 | 7.547 | -0.864 | -7.926 | 6.199 | -4.346 | -10.466 | 1.775 | -0.191 | -4.908 | 4.525 |
|  | Tryptophan | 1.013 | -3.705 | 5.730 | -1.456 | -5.538 | 2.626 | 1.788 | -5.681 | 9.256 | 0.355 | -5.959 | 6.669 |
|  | L-methionine | -0.767 | -5.683 | 4.149 | -0.707 | -5.248 | 3.833 | -8.056 | -15.746 | -0.365 | 0.656 | -5.551 | 6.864 |
|  | Citrulline | 1.624 | -8.406 | 11.654 | -1.282 | -9.216 | 6.653 | -3.196 | -18.996 | 12.604 | -0.399 | -5.378 | 4.580 |
|  | Sorbitol | -0.508 | -6.765 | 5.750 | -0.069 | -4.245 | 4.107 | -3.894 | -8.171 | 0.382 | 0.565 | -6.434 | 7.563 |
|  | Allantoin | 6.251 | -2.366 | 14.868 | 0.213 | -7.067 | 7.493 | 2.462 | -2.436 | 7.360 | 0.518 | -3.880 | 4.916 |
|  | Creatinine | -10.993 | -30.462 | 8.475 | 0.496 | -5.261 | 6.252 | -4.276 | -7.895 | -0.657 | 0.507 | -4.496 | 5.511 |
|  | deoxycytidine | -0.338 | -11.643 | 10.967 | -1.126 | -8.037 | 5.785 | 11.007 | -2.664 | 24.678 | 0.054 | -4.740 | 4.849 |
|  | Guanosine | -1.085 | -8.598 | 6.428 | -0.049 | -4.560 | 4.462 | -0.453 | -7.615 | 6.710 | -0.576 | -4.654 | 3.502 |
|  | Isobutyrylcarnitine | -4.504 | -18.426 | 9.418 | -0.120 | -5.735 | 5.494 | -5.641 | -16.357 | 5.076 | 0.426 | -6.516 | 7.368 |
|  | Xanthosine | -1.270 | -4.094 | 1.554 | -1.419 | -3.770 | 0.933 | 24.571 | 17.511 | 31.630 | -0.009 | -2.613 | 2.595 |
|  | Phosphoethanolamine | 1.388 | -6.368 | 9.144 | -0.971 | -7.436 | 5.495 | 3.861 | -1.632 | 9.353 | 0.378 | -5.257 | 6.014 |
|  | NAD | -2.562 | -27.059 | 21.934 | -1.346 | -11.193 | 8.501 | 14.652 | -6.089 | 35.394 | -0.683 | -13.554 | 12.187 |
|  | GABA | 0.665 | -4.339 | 5.670 | -1.584 | -7.533 | 4.364 | -3.691 | -9.840 | 2.459 | 0.034 | -5.224 | 5.292 |
|  | Sucrose | 0.855 | -4.000 | 5.709 | -1.267 | -7.705 | 5.170 | 1.428 | -4.011 | 6.867 | 0.672 | -3.522 | 4.866 |
|  | AMP | -0.954 | -12.947 | 11.039 | -0.985 | -7.928 | 5.959 | 5.021 | -6.664 | 16.706 | 0.453 | -4.395 | 5.301 |
|  | Dimethyl glycine | -0.204 | -4.338 | 3.931 | -1.320 | -5.077 | 2.438 | 16.401 | 6.147 | 26.654 | 0.039 | -4.432 | 4.510 |
|  | Guanidinoacetic acid | 8.150 | -2.337 | 18.637 | -0.677 | -4.255 | 2.901 | 4.741 | 0.548 | 8.934 | -0.599 | -6.674 | 5.476 |
|  | Cytidine | -0.153 | -4.246 | 3.939 | 1.080 | -3.934 | 6.095 | -4.308 | -8.832 | 0.217 | 0.161 | -5.616 | 5.937 |
|  | L-kynurenine | 0.300 | -4.260 | 4.860 | -1.574 | -6.550 | 3.402 | 5.872 | 2.581 | 9.163 | 0.373 | -5.609 | 6.356 |
|  | Spermidine | 0.691 | -4.900 | 6.281 | -0.443 | -4.062 | 3.176 | 0.356 | -5.632 | 6.344 | 1.269 | -6.834 | 9.373 |
|  | Taurocholic acid | 1.200 | -2.763 | 5.163 | 1.179 | -7.448 | 9.806 | -6.753 | -12.888 | -0.617 | 0.972 | -4.538 | 6.483 |
|  | Propionylcarnitine | 1.924 | -11.194 | 15.041 | -0.700 | -9.160 | 7.760 | 2.250 | -4.440 | 8.939 | 0.526 | -3.775 | 4.826 |
|  | Taurochenodeoxycholic acid | 2.519 | -2.210 | 7.248 | -1.890 | -11.786 | 8.006 | -1.686 | -6.308 | 2.937 | 0.288 | -4.829 | 5.404 |
|  | 2-aminoisobutyric acid | 0.931 | -4.774 | 6.636 | -0.693 | -5.189 | 3.804 | -0.582 | -14.749 | 13.585 | -1.091 | -7.224 | 5.041 |
|  | Glycocholic acid | 3.750 | -2.899 | 10.398 | -2.549 | -18.595 | 13.497 | -3.507 | -8.376 | 1.362 | -0.292 | -3.708 | 3.125 |
|  | Isovalerylcarnitine | 3.964 | -21.977 | 29.905 | 0.452 | -6.685 | 7.588 | -5.487 | -11.762 | 0.788 | 0.814 | -3.691 | 5.319 |
|  | Trimethylamine-N-oxide | 1.484 | -7.910 | 10.877 | -0.491 | -5.369 | 4.387 | 3.273 | -0.738 | 7.284 | 0.924 | -3.431 | 5.279 |
|  | Octanoylcarnitine | -0.397 | -9.593 | 8.800 | 0.775 | -3.572 | 5.122 | -5.066 | -8.969 | -1.163 | -0.545 | -8.241 | 7.151 |
|  | Decanoylcarnitine | -6.282 | -15.731 | 3.167 | 0.584 | -5.372 | 6.539 | -50.033 | -73.653 | -26.412 | 4.488 | -10.086 | 19.062 |
|  | Hexanoylcarnitine | -1.636 | -11.996 | 8.725 | 0.136 | -4.377 | 4.650 | -3.392 | -8.028 | 1.244 | -0.224 | -4.217 | 3.769 |
|  | Cytosine | 4.521 | 0.312 | 8.729 | -0.780 | -4.529 | 2.968 | 22.469 | 11.988 | 32.950 | 0.154 | -3.378 | 3.685 |
|  | 4-pyridoxic acid | -1.234 | -6.513 | 4.044 | -1.163 | -4.033 | 1.707 | 16.920 | 7.896 | 25.943 | -0.256 | -4.243 | 3.731 |
|  | Kynurenic acid | 1.462 | -2.650 | 5.573 | -1.508 | -5.116 | 2.100 | 7.571 | 2.742 | 12.400 | -0.240 | -7.443 | 6.962 |
|  | Adenine | 6.358 | -8.621 | 21.336 | -0.404 | -6.885 | 6.078 | -2.091 | -8.862 | 4.681 | -0.233 | -3.531 | 3.064 |
|  | Normetanephrine | 5.158 | -3.648 | 13.963 | -1.707 | -7.542 | 4.129 | 1.815 | -2.002 | 5.632 | -0.812 | -6.815 | 5.191 |
