# Supplementary material for: Interactive effects of aging and aerobic capacity on energy metabolism–related metabolites of serum, skeletal muscle, and white adipose tissue
Source: GeroScience. 2021 Jun 5;43(6):2679–91. doi: 10.1007/s11357-021-00387-1 (PMC8602622; doi:10.1007/s11357-021-00387-1)
Supplement: Supplementary file 7 — (DOCX 25 kb) [file 11357_2021_387_MOESM6_ESM.docx]

**Supplementary Table 5**. Design-controlled skew and kurtosis estimates for metabolites after most optimal transformation (Tf).

| **Serum**  **Metabolite** | **Skew** | **Kurtosis** | **λ^a^** | **Tf** |
| --- | --- | --- | --- | --- |
| Lysine | -0.30 | 0.43 |  | N |
| Alanine | -0.09 | 0.48 |  | N |
| Glutamine | -1.32 | 1.68 | -0.72 | B |
| Betaine | 0.48 | 1.41 | -1.35 | B |
| Creatine | 1.83 | 4.99 | -0.39 | B* |
| Taurine | -0.95 | 1.42 |  | N |
| Glyceraldehyde | 1.59 | 3.32 | -0.09 | B* |
| Serine | 0.26 | 0.05 |  | N |
| Threonine | 1.02 | 1.27 |  | N |
| Allantoin | 0.21 | -0.83 |  | N |
| Hippuric.acid | -0.36 | 0.87 |  | N |
| Leucine | -0.18 | -0.42 |  | N |
| Chenodeoxycholic.Acid | -0.05 | -0.90 |  | L |
| Valine | 0.27 | -0.89 |  | N |
| L.Glutamic.Acid | 0.58 | -0.22 |  | N |
| Glycine | 0.53 | 0.85 |  | N |
| Citrulline | 0.17 | 0.08 |  | L |
| Proline | -0.01 | 1.23 |  | N |
| Choline | -0.45 | 1.32 |  | N |
| Ornithine | -0.38 | 0.49 |  | N |
| Hydroxyproline | -0.33 | 0.03 | 0.39 | B |
| Histidine | -0.32 | -0.57 |  | N |
| Isoleucine | -0.74 | 0.59 | 1.87 | B |
| Tryptophan | -0.17 | 0.15 |  | N |
| Myoinositol | -0.26 | -0.41 |  | N |
| Tyrosine | 0.10 | -0.44 |  | N |
| Phenylalanine | -1.06 | 2.84 | 1.04 | B* |
| Asparagine | 0.36 | -0.25 |  | N |
| Carnitine | 0.52 | 0.66 |  | N |
| Acetoacetic.acid | -0.14 | -0.19 |  | N |
| Creatinine | 0.19 | -0.83 |  | N |
| Succinate | -0.44 | 0.22 | -0.36 | B |
| L.Methionine | -1.04 | 0.48 |  | L |
| deoxycytidine | -0.31 | 0.52 |  | N |
| Arginine | 0.84 | -0.16 |  | N |
| Aspartate | 0.08 | 0.28 | 0.32 | B |
| Homocysteine | -0.12 | 0.07 |  | N |
| Acetylcarnitine | 0.21 | 0.26 | -0.92 | B |
| Uracil | -0.02 | -0.73 | 0.21 | B |
| Niacinamide | 0.59 | 0.48 |  | N |
| D.Glucuronic.acid | 0.36 | -0.20 |  | L |
| D.Ribose.5.phosphate | 0.14 | -0.20 |  | L |
| Sorbitol | -0.09 | -0.41 | -0.66 | B |
| Phosphoethanolamine | 0.80 | 1.72 |  | L |
| DimethylGlycine | 0.34 | 3.35 |  | N* |
| X2.Aminoisobutyric.acid | -0.22 | -0.44 | 0.14 | B |
| Spermidine | -0.31 | 0.26 | 0.53 | B |
| Pantothenic.Acid | 0.38 | 0.73 |  | N |
| Aminodipic.Acid | 0.49 | 0.08 |  | N |
| Carnosine | -0.21 | -0.38 |  | L |
| Asymmetric.dimethylarginine | 0.73 | 0.66 |  | N |
| Guanidinoacetic.Acid | 0.44 | -0.33 |  | N |
| Taurocholic.Acid | -0.45 | -0.25 | -0.50 | B |
| L.Kynurenine | -0.12 | -0.71 |  | L |
| Trimethylamine.N.Oxide | 0.26 | -0.75 |  | N |
| Symmetric.dimethylarginine | 0.13 | -0.39 |  | N |
| X2.deoxyuridine | 0.35 | -0.71 |  | L |
| Sucrose | 0.14 | -0.75 | -1.77 | B |
| Xanthosine | 0.80 | -0.39 |  | N |
| IMP | 0.84 | 0.99 | -0.68 | B |
| Propionylcarnitine | 0.30 | 0.12 |  | N |
| X5.Hydroxyindole.3.acetic.acid | 0.53 | 0.28 |  | L |
| Glycocholic.Acid | -0.42 | 0.93 | 0.12 | B |
| Isobutyrylcarnitine | 0.00 | -0.55 |  | L |
| Taurochenodeoxycholic.Acid | 0.04 | -0.72 | -0.24 | B |
| Cystathionine | 0.82 | 0.48 |  | N |
| Cytidine | 0.28 | 0.15 | -0.18 | B |
| Cholic.Acid | 0.57 | -0.83 |  | N |
| GABA | 0.25 | -0.06 | -0.06 | B |
| Gamma.Glutamylcysteine | -0.04 | 0.43 | -0.79 | B |
| Octanoylcarnitine | 0.25 | -0.23 |  | L |
| Kynurenic.Acid | 0.80 | 0.75 |  | N |
| Isovalerylcarnitine | 0.22 | -0.35 |  | N |
| AMP | 0.33 | -0.63 | -0.75 | B |
| Decanoylcarnitine | -0.11 | -1.25 | 0.27 | B |
| X4.Pyridoxic.Acid | 0.61 | 0.18 |  | N |
| Folic.Acid | -0.29 | 0.52 |  | N |
| Inosine | 0.31 | -0.60 | -0.66 | B |
| NAD | 0.77 | 0.75 | 0.05 | B |
| Nicotinic.Acid | 0.29 | 0.26 | -0.19 | B |
| Adenosine | -0.28 | -0.13 | -0.32 | B |
| Hexanoylcarnitine | 0.02 | 0.30 |  | L |
| Xanthine | -0.06 | -0.51 |  | L |
| X1.methylhistamine | 0.07 | 0.27 | -0.06 | B |
| Cytosine | 0.10 | -0.85 |  | N |
| Adenine | 0.14 | -0.25 |  | N |

^a^The parameter of the BoxCox transformation.

Note. N = no transformation, L = log-transformation, B = BoxCox-transformation, *high kurtosis despite any transformation.

**Table**. Design-controlled skew and kurtosis estimates for metabolites after most optimal transformation (Tf).

| **Muscle**  **Metabolite** | **Skew** | **Kurtosis** | **λ^a^** | **Tf** |
| --- | --- | --- | --- | --- |
| Alanine | 0.00 | -0.09 | 0.63 | N |
| Taurine | -0.25 | 0.35 | 0.64 | L |
| Hydroxyproline | -0.02 | 0.92 | 0.41 | N |
| Creatine | -0.48 | -0.32 | -1.77 | B |
| Glutamine | -0.29 | 0.65 | 0.21 | N |
| Acetoacetic.acid | 0.28 | -0.43 | 0.42 | N |
| Glycine | -1.16 | 2.56 | 0.36 | B* |
| Niacinamide | -0.13 | -0.66 | 1.30 | N |
| Carnitine | 0.20 | 3.77 | 1.00 | N* |
| Serine | 0.67 | 0.17 | 0.42 | N |
| Threonine | 0.63 | -0.03 | 1.04 | N |
| Acetylcarnitine | -0.28 | -0.42 | 1.28 | N |
| L.Glutamic.Acid | 0.21 | -1.26 | 0.11 | B |
| Choline | 0.17 | 0.13 | -0.13 | N |
| Glutathione | -0.37 | 0.06 | -0.64 | B |
| Creatinine | -0.67 | 4.46 | -0.91 | N* |
| Proline | 0.43 | 0.26 | 0.31 | N |
| Hypoxanthine | 1.05 | 2.34 | -0.07 | N* |
| Valine | 0.34 | 0.02 | 0.38 | N |
| Succinate | -0.03 | -0.88 | 0.27 | N |
| Leucine | -1.14 | 2.88 | 1.03 | N* |
| Inosine | 0.17 | -0.55 | -0.01 | B |
| Glyceraldehyde | 0.58 | 0.84 | -0.31 | N |
| Allantoin | -0.95 | 1.17 | -0.25 | B |
| Betaine | -0.28 | -0.49 | 0.03 | L |
| Citrulline | 0.08 | -0.93 | -0.23 | B |
| Asparagine | 0.37 | 0.28 | -0.24 | B |
| Tyrosine | 0.28 | -0.11 | -0.98 | L |
| Aspartate | 0.88 | 0.75 | -0.32 | B |
| Isoleucine | -0.03 | -0.04 | -0.38 | N |
| Ornithine | -0.01 | -0.02 | -0.71 | B |
| Phenylalanine | -0.07 | 0.21 | -0.57 | N |
| L.Methionine | -0.48 | 0.67 | 0.61 | N |
| Uracil | 0.00 | -0.41 | -0.44 | B |
| Tryptophan | -0.10 | -0.58 | -1.46 | B |
| Xanthine | 0.44 | 0.55 | 0.17 | B |
| IMP | -0.23 | -0.20 | -0.01 | L |
| Pantothenic.Acid | 0.71 | 0.53 | 0.29 | N |
| Chenodeoxycholic.Acid | 1.23 | 3.88 | -0.02 | B* |
| Sorbitol | 0.25 | 0.34 | -0.70 | N |
| Hippuric.acid | -0.25 | 0.93 | 0.37 | N |
| deoxycytidine | -0.52 | 2.75 | -0.18 | L* |
| Taurocholic.Acid | 0.26 | 0.22 | 1.11 | N |
| X2.Aminoisobutyric.acid | 0.24 | -0.70 | -0.36 | L |
| Cytidine | -0.01 | 1.28 | 1.47 | B |
| Octanoylcarnitine | 0.32 | 0.01 | -0.51 | L |
| Phosphoethanolamine | 0.14 | -1.19 | 0.00 | L |
| Isobutyrylcarnitine | 0.40 | -0.31 | -0.64 | L |
| Folic.Acid | 0.36 | -0.18 | 0.15 | B |
| Hexanoylcarnitine | 0.08 | -0.66 | -0.76 | B |
| Decanoylcarnitine | 0.69 | 0.43 | -0.37 | L |
| Guanidinoacetic.Acid | 0.50 | 0.38 | -0.08 | N |
| DimethylGlycine | -0.58 | -0.05 | 0.82 | L |
| Sucrose | 0.01 | 0.27 | -0.72 | B |
| L.Kynurenine | 0.69 | -0.43 | -0.23 | N |
| Trimethylamine.N.Oxide | -0.12 | -0.47 | 0.32 | N |
| Propionylcarnitine | -0.71 | 1.21 | -0.89 | B |
| NAD | 0.70 | 2.36 | 0.12 | L* |
| Isovalerylcarnitine | 0.55 | 0.22 | -0.13 | N |
| GABA | -0.09 | -0.20 | -0.06 | B |
| Spermidine | 0.15 | -0.10 | -0.34 | B |
| Glycocholic.Acid | 0.32 | -0.30 | 1.05 | N |
| X2.deoxyuridine | 0.58 | -0.15 | -0.01 | N |
| AMP | 0.25 | -0.48 | -0.11 | L |
| Adenosine | 0.00 | -0.66 | -0.16 | L |
| Adenine | 0.38 | 0.62 | -0.20 | B |
| Cytosine | 0.67 | 0.79 | 0.25 | N |
| Kynurenic.Acid | 0.18 | -0.46 | -0.06 | B |
| Taurochenodeoxycholic.Acid | 0.12 | -0.61 | -0.21 | L |
| Normetanephrine | 0.49 | 0.19 | 0.04 | L |
| X4.Pyridoxic.Acid | 0.84 | 0.97 | 0.05 | L |

^a^The parameter of the BoxCox transformation.

Note. N = no transformation, L = log-transformation, B = BoxCox-transformation, *high kurtosis despite any transformation.

**Table**. Design-controlled skew and kurtosis estimates for metabolites after most optimal transformation (Tf).

| **WAT**  **Metabolite** | **Skew** | **Kurtosis** | **λ^a^** | **Tf** |
| --- | --- | --- | --- | --- |
| Taurine | -0.69 | -0.25 | 1.55 | L |
| Alanine | 0.15 | -0.81 | -0.82 | B |
| Choline | -0.17 | -0.85 | 0.91 | B |
| Acetoacetic.acid | 0.03 | -0.87 | -0.17 | L |
| Myoinositol | -0.43 | 0.31 | 0.55 | N |
| Glutathione | -0.14 | -0.42 | 0.88 | N |
| Hypoxanthine | -0.21 | -0.49 | 0.29 | B |
| Creatine | 0.19 | 0.17 | -0.35 | B |
| Hydroxyproline | -0.01 | -0.32 | 0.00 | L |
| Glutamine | 0.06 | -0.84 | -0.03 | N |
| Glycine | 0.28 | -0.30 | 1.28 | N |
| L.Glutamic.Acid | 0.56 | -0.21 | 0.28 | N |
| Serine | 0.59 | 0.19 | -0.34 | N |
| Succinate | -0.09 | 0.99 | 0.56 | B |
| Threonine | 0.21 | 0.39 | 0.44 | L |
| Inosine | 0.06 | -0.48 | 0.56 | N |
| Ornithine | 0.12 | -0.20 | 0.76 | N |
| Niacinamide | -0.58 | 0.02 | 1.04 | L |
| Chenodeoxycholic.Acid | -0.89 | 1.51 | -0.16 | B |
| Proline | 0.08 | -0.89 | 0.87 | N |
| Leucine | 0.39 | 0.26 | 0.09 | N |
| Valine | 0.21 | 0.10 | 0.35 | N |
| Uracil | 0.50 | -0.49 | 0.61 | N |
| Arginine | -0.38 | 1.80 | -0.54 | B |
| Adenosine | -1.33 | 3.06 | 0.07 | B* |
| Betaine | 0.63 | -0.28 | 0.61 | N |
| Aspartate | 0.25 | -0.07 | 0.77 | N |
| Tyrosine | -0.21 | 0.06 | 0.36 | L |
| Xanthine | 0.25 | -0.59 | 0.67 | N |
| Asparagine | 0.19 | -0.88 | 0.46 | N |
| Hippuric.acid | 0.09 | -0.04 | -0.03 | B |
| Carnitine | 0.55 | -0.66 | 0.57 | N |
| Acetylcarnitine | 0.55 | -0.60 | -1.04 | L |
| Pantothenic.Acid | 0.41 | -0.95 | 0.16 | N |
| Phenylalanine | 0.67 | -0.22 | 0.19 | N |
| Tryptophan | 0.21 | -0.57 | 0.70 | N |
| L.Methionine | -0.77 | 0.28 | 0.98 | L |
| Citrulline | 0.16 | -0.70 | 0.28 | N |
| Sorbitol | 0.14 | -0.98 | 0.41 | N |
| Allantoin | -0.09 | -0.90 | 0.54 | N |
| Creatinine | 0.86 | 0.76 | -0.22 | N |
| deoxycytidine | 0.02 | 0.00 | -0.55 | B |
| Guanosine | 0.23 | -0.73 | 0.44 | N |
| Isobutyrylcarnitine | -0.05 | -0.26 | 0.29 | B |
| Xanthosine | 0.05 | -0.64 | 0.68 | N |
| Phosphoethanolamine | 0.41 | -0.62 | 0.42 | N |
| NAD | -0.75 | 1.06 | -0.02 | B |
| GABA | 0.01 | -0.49 | -0.14 | B |
| Sucrose | 0.54 | -0.72 | -0.27 | N |
| AMP | 0.00 | -0.14 | -0.13 | L |
| DimethylGlycine | 0.54 | 0.79 | 0.38 | N |
| Guanidinoacetic.Acid | 0.04 | -0.27 | -0.06 | B |
| Cytidine | 0.60 | -0.56 | -0.96 | L |
| L.Kynurenine | 0.36 | -0.63 | -0.24 | L |
| Spermidine | 0.20 | 0.55 | 0.14 | B |
| Taurocholic.Acid | 0.28 | -0.75 | -0.65 | B |
| Propionylcarnitine | 0.28 | -0.45 | 0.34 | N |
| Taurochenodeoxycholic.Acid | 0.32 | -1.12 | -0.23 | L |
| X2.Aminoisobutyric.acid | 0.46 | -0.44 | 0.20 | N |
| Glycocholic.Acid | -0.60 | -0.18 | 0.14 | L |
| Isovalerylcarnitine | 0.24 | -0.93 | -0.07 | N |
| Trimethylamine.N.Oxide | 0.54 | -0.83 | 0.06 | N |
| Octanoylcarnitine | 0.72 | -0.71 | -0.82 | L |
| Decanoylcarnitine | 0.27 | -0.03 | 0.34 | L |
| Hexanoylcarnitine | 0.76 | 0.09 | -0.65 | L |
| Cytosine | 0.02 | -0.72 | 0.08 | L |
| X4.Pyridoxic.Acid | -0.01 | -0.35 | 0.14 | L |
| Kynurenic.Acid | 0.11 | -0.81 | -0.55 | B |
| Adenine | 0.53 | -0.51 | 0.01 | N |
| Normetanephrine | 0.55 | -0.14 | 0.34 | N |

^a^The parameter of the BoxCox transformation.

Note. N = no transformation, L = log-transformation, B = BoxCox-transformation, *high kurtosis despite any transformation.
