# Supplementary material for: Interactive effects of aging and aerobic capacity on energy metabolism–related metabolites of serum, skeletal muscle, and white adipose tissue
Source: GeroScience. 2021 Jun 5;43(6):2679–91. doi: 10.1007/s11357-021-00387-1 (PMC8602622; doi:10.1007/s11357-021-00387-1)
Supplement: Supplementary file 8 — (DOCX 15 kb) [file 11357_2021_387_MOESM7_ESM.docx]

**Technical Supplement**

**Details of univariate linear regression models**

The models include factor variables *A* as the age-group (young / old) and *C* as the aerobic capacity (high / low). For rat *i* = 1, …, *n* ja metabolite *j* = 1, …, *J*, the age and aerobic capacity group differences in metabolites were based on the linear model:

$m_{i(j)}=\hat{m}_{i(j)}+\epsilon_{i}$,

where $m_{i(j)}$ is the value for metabolite *j* of rat *i*, $\epsilon_{i}$ is the residual term, and the expected value of metabolite, $\hat{m}_{i(j)}$, is given by:

$\hat{m}_{i(j)}=\alpha_{M_{(j)}}+G_{A}A_{i}+G_{C}C_{i}+G_{AC}A_{i}C_{i}$,

The expectation includes $\alpha_{M_{(j)}}$ as the intercept term in a regression model involving metabolite *j*, and unstandardized regression coefficients for main effects of age and aerobic capacity, *G_A_* and *G_C_*, respectively, and the interaction term for age and aerobic capacity, *G_AC_*. The marginal means for metabolite *j* in age-aerobic capacity groups resulting from the model can be computed from the regression coefficients as follows:

$\hat{\upsilon}_{j(C=Low,A=Old)}=\alpha_{M_{(j)}}$,

$\hat{\upsilon}_{j(C=High,A=Old)}=\alpha_{M_{(j)}}+G_{C}$,

$\hat{\upsilon}_{j(C=Low,A=Young)}=\alpha_{M_{(j)}}+G_{A}$,

and

$\hat{\upsilon}_{j(C=High,A=Young)}=\alpha_{M_{(j)}}+G_{A}+G_{C}+G_{AC}$.

The standard errors for the marginal means were computed using the delta-method and significance of marginal mean differences were computed using the Wald-test.

Univariate association of a metabolite *j* with running speed was based on the linear model:

$s_{i(j)}=\hat{s}_{i(j)}+\varepsilon_{i}$,

where $s_{i(j)}$ is the running speed of rat *i* in a model involving metabolite *j*, $\varepsilon_{i}$ is the residual term, and the expected value of running speed, $\hat{s}_{i(j)}$, is given by:

$$\hat{s}_{i(j)}=\alpha_{S_{(j)}}+B_{A}A_{i}+B_{C}C_{i}+B_{M}M_{i\left( j \right)}+$$

$B_{AC}A_{i}C_{i}+B_{AM}A_{i}M_{i(j)}+B_{CM}C_{i}M_{i(j)}+B_{ACM}A_{i}C_{i}M_{i(j)}$,

The expectation includes $\alpha_{S_{(j)}}$ as the intercept term, *M_i_*_(_*_j_*_)_ is rat *i*’s value for metabolite *j*, the unstandardized main effects regression coefficients *B_A_*, *B_C_*, *B_M_* for age, aerobic capacity, and metabolite, respectively, two-variable interaction terms, *B_AC_*, *B_AM_*, *B_CM_* and the three-variable interaction term *B_ACM_*. The marginal regression coefficients for running speed regressed on the metabolite in age-aerobic capacity groups resulting from the model are given by:

$\nu_{j(C=Low,A=Old)}=B_{M}$,

$\nu_{j(C=High,A=Old)}=B_{M}+B_{CM}$,

$\nu_{j(C=Low,A=Young)}=B_{M}+B_{AM}$,

and

$\nu_{j(C=High,A=Young)}=B_{M}+B_{AM}+B_{CM}+B_{ACM}$.

The standard errors for the marginal means were computed using the delta-method and significance of marginal mean differences were computed using the Wald-test.
